# Cytolytic CD4^+^ and CD8^+^ Regulatory T-Cells and Implications for Developing Immunotherapies to Combat Graft-Versus-Host Disease

**DOI:** 10.3389/fimmu.2022.864748

**Published:** 2022-04-12

**Authors:** Sara Bolivar-Wagers, Jemma H. Larson, Sujeong Jin, Bruce R. Blazar

**Affiliations:** Department of Pediatrics, Division of Blood & Marrow Transplant & Cellular Therapy, University of Minnesota, Minneapolis, MN, United States

**Keywords:** regulatory T-cell, tTreg, pTreg, iTreg, cytotoxic, GVHD, CAR T-cells

## Abstract

Regulatory T-cells (Treg) are critical for the maintenance of immune homeostasis and tolerance induction. While the immunosuppressive mechanisms of Treg have been extensively investigated for decades, the mechanisms responsible for Treg cytotoxicity and their therapeutic potential in regulating immune responses have been incompletely explored and exploited. Conventional cytotoxic T effector cells (Teffs) are known to be important for adaptive immune responses, particularly in the settings of viral infections and cancer. CD4+ and CD8+ Treg subsets may also share similar cytotoxic properties with conventional Teffs. Cytotoxic effector Treg (cyTreg) are a heterogeneous population in the periphery that retain the capacity to suppress T-cell proliferation and activation, induce cellular apoptosis, and migrate to tissues to ensure immune homeostasis. The latter can occur through several cytolytic mechanisms, including the Granzyme/Perforin and Fas/FasL signaling pathways. This review focuses on the current knowledge and recent advances in our understanding of cyTreg and their potential application in the treatment of human disease, particularly Graft-versus-Host Disease (GVHD).

## Introduction

Regulatory T cells (Treg) play a complex multifaceted role in maintaining immune homeostasis and promoting tolerance at steady state. Treg are widely regarded to engage in various suppressive mechanisms directed against T-cells and antigen presenting cells (APC). Since their discovery, CD4^+^CD25^+^ T-cells have been found to protect against autoimmune disease (1) and are known to be critical for tolerance against alloresponses *in vivo* (2) including bone marrow and solid organ transplantation tolerance in allogeneic murine recipients (3–5). Adoptive transfer of Treg with alloreactive T-cells have shown efficacy in limiting the Graft-Versus-Host Disease (GVHD) response in preclinical transplant models, and Graft-versus-Leukemia (GVL) responses have by-and-large, but not uniformly, remained intact (6–8). Clinical trials have demonstrated that Treg infusion during allogeneic hematopoietic stem cell transplantation (alloHSCT) can reduce and treat GVHD (9–14). Decisive conclusions on post-transplant relapse rates await randomized trials. To date, current studies observing high-risk acute leukemia adult patients treated by haploidentical transplantation with an infusion of Foxp3^+^ Treg four days prior to T-cells have shown similar reductions in GVHD occurrence and severity as previous studies and the cumulative incidence of relapse was significantly lower than their historical controls (11–13). This balance between ameliorating GVHD while preserving GVL continues to be a major consideration for the development of effective GVHD treatments. Recent data has come to light that subsets of cytotoxic effector Treg (cyTreg) may have a unique application here and are capable of suppressing GVHD while effectively preserving GVL activity (8, 15–17).

Studies dating back to the early 2000s demonstrated that Treg engage in cytolytic activities. While cytolysis is a well characterized mechanism of conventional cytotoxic T effector cells (Teffs) and natural killer (NK) cells, it was surprising to find that Treg also engaged in directed killing of target cells to suppress immune responses while maintaining traditional suppressive capabilities (18, 19). Before cytolysis was directly ascribed as a mechanism of suppression by Treg, cytolysis had been reported as a critical pathway for immune homeostasis as its been documented that mutations in killing pathways are associated with many autoimmune and inflammatory diseases, including autoimmune lymphoproliferative syndrome (ALPS) (20–23) and Griscelli’s syndrome (24–26). There is now a growing repository of data supporting the important role of cytotoxicity in cyTreg for immune regulation and disease control. In this review, we discuss the current and growing knowledge of cyTreg, the directed killing and immunosuppressive mechanisms that drive their function, and their potential clinical applications for the treatment of human disease, including cancer, inflammatory disease and GVHD.

### Mechanisms of cyTreg Mediated Suppression and Cytotoxicity

**Figure 1:**
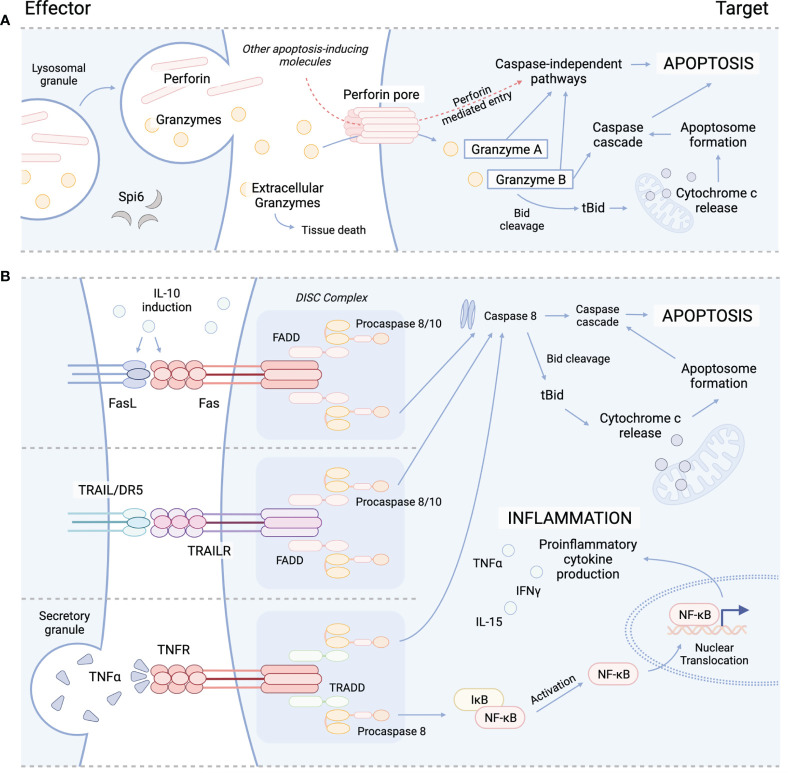
Cytolytic mechanisms utilized by cyTreg to modulate immune responses. **(A)** Granzyme perforin pathway. **(B)** Fas/FasL pathway (top), TRAIL/TRAILR pathway (middle), TNFα/TNFR pathway (bottom). Spi6, Serpin protease inhibitor 6; TRAIL, tumor necrosis factor-related apoptosis-inducing ligand; TRAILR, tumor necrosis factor-related apoptosis-inducing ligand receptor; DR5, death receptor; TNFα, tumor necrosis factor alpha; TNFR, TNFR, tumor necrosis factor receptor; Bid, BH3 interacting-domain death agonist; tBid, truncated Bid; FADD, Fas-associated protein with death domain; TRADD, TNFRSF1A associated *via* death domain; NF-kB, nuclear factor kappa B; DISC, death-inducing signaling complex.

The primary function of Treg is to maintain immune homeostasis and self-tolerance by modulating the activity of effector lymphocyte populations. Traditionally, both CD4^+^ and CD8^+^ Treg have been recognized to impart immunosuppressive effects through contact dependent and independent mechanisms. For example, CTLA-4 expression by Treg is recognized as an integral marker of contact dependent immunosuppression. CTLA-4 is known to inhibit T-cell activation and expansion by both directly engaging with CD28 on the surface of T-cells to block co-stimulation, and by cleaving CD80/CD86 from the surface of APC to further inhibit APC/T-cell interactions (27–31). In addition to contact dependent pathways, Treg have also been shown to suppress T-cell activity through the release of soluble factors, such as IL-10 and TGF-β. IL-10 is produced by several immune cell populations, including Treg and has been shown to be important for preventing mucosal inflammation and autoimmunity (32–34). IL-10 is known to suppress the activity of T-cells by inhibiting the APC expression of MHC class II and CD80/86 and the production of proinflammatory cytokines (IL1α, IL1β, IL-12, IL-18, TNFα) and chemokines (MCP1, MCP5, RANTES, IP-10, IL-8, and MIP-2), as well as impeding T-cell proliferation and cytokine production (IL-2, IFNγ, IL-4, IL-5, TNFα). Similarly, TGF-β production has also been shown to be important in modulating inflammation (35–37), and the contact-dependent function of TGF-β has been well-described to orchestrate the induction and maintenance of peripheral Treg (pTreg) (38, 39), induced Treg (iTreg) (38, 40), and invariant natural killer T-cells (iNKT) (41, 42). Several studies have highlighted the suppressive activity of Treg-derived TGF-β, which has been reported to inhibit both NK and Teff cytotoxic activity (43–45). Interestingly, both IL-10 and TGF-β signaling have also been reported to play a role in cytotoxicity. While TGF-β has been shown to drive the induction of CD103 expression in CD8^+^ iTreg, which in some circumstances has been shown to be a simultaneously cytotoxic and immunosuppressive T-cell subset (46), IL-10 has also demonstrated cytolytic characteristics during systemic lupus erythematosus (SLE) (47–49). IL-10 produced by B cells and monocytes can induce Fas/FasL expression on cell surfaces of various immune cells, including T-cells to cause apoptosis *via* caspase 8 activation (47, 48). IL-10 induced Fas/FasL pathway is important for disease control and has otherwise been an under reported functional mechanism of IL-10 signaling (**Figure 1**) (50–52). Similarly, IL-10 has also been shown to induce the production of cytotoxic enzymes and IFNγ in CD8^+^ T-cells, and, in conjunction with IL-2, differentiation of cytotoxic T-cells from their precursors, demonstrating a dual function of IL-10 signaling in immune regulation (53, 54).

Treg can be classified into subtypes based on their phenotypic profiles, cytokine production, and expression of lineage defining transcription factors such T-bet and RORγt (55). With the use of advanced sequencing technologies, we are expanding our understanding of the similarities and unique aspects of Treg subtypes based on their transcriptome (56, 57). Höllbacher et al. (2020) (58) performed RNA sequencing on Treg and found that subtypes clustered together, although with notable differences, suggesting lower diversity amongst Treg as compared to CD4^+^ T helper cells (Th). For example, expression of IL-10 was limited to only a few Treg subtypes which included Th1-like Treg and Th17-like Treg. Th1-like Treg also had the highest expression of the coinhibitory receptors *TIM3* and *LAG3*, and the cytolytic molecules *GZMA* and *GZMB* (58). In contrast, other genes associated with Treg suppressive function such as *CTLA4*, *PDCD1*, *TIGIT*, and *PRF1*, were not preferentially expressed by any Treg subtype (58). Furthermore, single cell RNA sequencing demonstrates Treg clustering within lymphoid tissues into central versus effector Treg populations, and within non-lymphoid tissues into multiple other Treg populations that represent tissue adaptation and local immune control (56). For example, in the colon of healthy mice there were three clusters of Treg, two which expressed genes associated with Th2 and immunoregulatory and immune suppressive Th3 lineages (56). These findings suggest each Treg subset may have a specialized mechanism to modulate specific types of immune responses, and that some may have a greater potential to engage in cytolytic mechanisms.

Several subsets of CD4^+^ and CD8^+^ Treg have been reported to engaged in directed killing of target cells through perforin and granzyme mechanisms. Perforin is a glycoprotein that polymerizes to form channels within target cell membranes (59, 60). These channels allow for free and non-selective transport of ions, water, and other molecules including pro-apoptotic granzymes, which disrupts cell homeostasis and cause cell death (59, 61). While Teff and NK cells are major sources of perforin and granzymes, which are released from cytoplasmic granules upon recognition of target cell (62), cyTreg have also been shown to produce perforin and granzymes (**Figure 1**) (18, 19, 27, 63). Granzymes are highly conserved serine proteases that make up the majority of the cytoplasmic granules of Teff and NK cells. While NK cells constitutively express and store granzymes, other T-cell subsets (i.e. CD4^+^ T-cell, CD4^+^ Treg) must be activated to produce granzymes (64, 65).

Granzymes maintain both cytotoxic and non-cytotoxic mechanisms to regulate immune responses. Ten granzymes have been discovered in mice, of which five are known in humans; these differ in their primary substrate specificities within target cells (66). Granzyme A (GzA) and B (GzB) are the most abundant and therefore the most frequently studied. GzA activates caspase-independent programmed cell death through cleavage of intracellular substrates, including mitochondrial complex I substrate NDUFS3 and precursor IL-1β (**Figure 1**) (67–70). GzB promotes apoptosis through the BH3-interacting domain death agonist (Bid), a proapoptotic Bcl-2 family member, by inducing mitochondrial permeabilization or direct proteolysis and activation of caspases (**Figure 1**) (71, 72). Though yet to be studied in Treg, special attention should be brought to the formation of supramolecular attack particles (SMAPs) by Teff (73) and NK cells (74) that allow for hours of sustained killing. Cytotoxic T-cell SMAPs are multiprotein complexes assembled from over 285 different proteins including cell adhesion molecules, chemokines, cytokines, and cytolytic perforin, granzymes, and galectin-1 stored within secretory lysosomes of cytolytic lymphocytes (73, 74). SMAPs are contained within a shell rich in glycoproteins, including perforin, granzymes and thrombospondin-1, that interact with target cells to theoretically allow autonomous killing by SMAPs, promoting killing during interactions that are transient or less precise (73).

While many of these cytotoxic mechanisms are well described in Teff and NK cells, there are important roles for these functional pathways in cyTreg function, which we discuss throughout this review. Although the role of many of these pathways in cyTreg remain unclear, there appears to be an intricate system of regulation for cyTreg to engage in killing and/or immunosuppressive functions *in vitro* and *in vivo*.

## CD4^+^ Treg Engage in Cytolysis to Regulate Immune Responses

CD4^+^CD25^+^Foxp3^+^ Treg are essential for maintaining immune homeostasis, preventing autoimmunity, and promoting tolerance (75). There are three major subsets of CD4^+^ Treg: thymic derived Treg (tTreg), Treg that are induced in the periphery from CD4^+^Foxp3^neg^ conventional T-cells (pTreg), and Treg generated *in vitro* from CD4^+^Foxp3^neg^ conventional T-cells using TGF-β and IL-2 (iTreg) (76). Treg have been reported to engage in multiple contact-dependent and independent mechanisms. These mechanisms can directly suppress immune cells or function indirectly by modulating APC and/or generating an anti-inflammatory milieu (77–79). For example, Treg can modulate APC through CTLA-4, suppress multiple cell types through secretion of anti-inflammatory cytokines (IL-10, TGF-β, IL-35), suppress T-cells *via* IL-2 consumption, use cytolytic pathways to kill T-cells or APC, and generate immunosuppressive environments through adenosine production *via* the ectoenzymes CD39 and CD73 (77) (**Table 1**).

**Table 1:** Immunosuppressive and cytotoxic killing mechanisms used by CD4^+^ Treg subsets.

Treg Subtype	Source/Origin	Key Markers	Organism	Functional Mechanisms	References
CD4^+^ tTreg	Thymus derived	Foxp3^+^, CD4^+^, CD25^hi^, CD127^lo^	Mouse	GzB dependent killing +/- perforin dependent killing TRAIL/DR5 dependent killing IL-10 mediated suppression CTLA-4 mediated suppression IL-2 deprivation CD39/CD73 adenosine mediated suppression	(80–91)
			Human	Perforin dependent killing Partially GzB dependent killing IL-10 mediated suppression CTLA-4 mediated suppression IL-2 deprivation	(18, 92–98)
CD4 pTreg	Peripherally induced	Foxp3^+^, CD25^hi^, CD127^lo^ often helios neg, Nrp1^neg^	Mouse	Fas/FasL dependent killing GzB dependent killing Perforin dependent killing	(99–101)
CD4 Tr1	Peripherally induced	Foxp3^neg^	Mouse	GzB dependent killing IL-10 mediated suppression	(15, 102, 103)
			Human	GzB dependent killing Perforin dependent killing IL-10 and TGF-β mediated suppression	(19, 103–105)

GzB, granzyme B; TRAIL/DR5, tumor necrosis factor related apoptosis ligand (TRAIL)/death receptor 5 (DR5) pathway.

Symbols: +/- pathway or marker is shown to be intermittently applicable or inconsistently reported between multiple studies.

While mouse studies have provided insights into the various mechanisms available for CD4^+^ Treg, the importance of these pathways in regulating immune tolerance is further supported in clinical reports of patients with inborn errors of immunity. For example, patients with *CTLA-4* haploinsufficiency experience immune dysregulation and deficiency with a spectrum of clinical manifestations that can include lymphoproliferation and autoimmunity targeting multiple organs such as the lungs, gastrointestinal tract, brain, bone marrow, kidney, hypophysis, and thyroid (92, 93). Importantly, these patients had either normal or elevated numbers of Treg, but CTLA-4 protein expression was significantly decreased on Treg and this was associated with impaired Treg suppressive function (92, 93). Patients with mutations in the Treg master transcription factor *FOXP3* that preclude CD4^+^ Treg generation or function experience immune dysregulation, polyendocrinopathy, enteropathy, X-linked (IPEX) syndrome with onset in early life and a high mortality rate (106–109). Treg have constitutive expression of the alpha subunit (CD25, or IL-2RA) of the trimeric IL-2R which allows them to uptake IL-2 more readily during an immune response and engage in a positive feedback cycle whereby IL-2 promotes Foxp3 expression in Treg (110). Patients with mutations in *IL2RA* can have severe enteritis, viral infection susceptibility, pronounced lymphoproliferation, autoimmunity, and an IPEX-like syndrome (94, 95, 111) (94, 95, 106–109). IL-10 signaling has been particularly important for tolerance induction in the gut, as patients with loss of function mutations in IL-10 or IL-10R develop very early onset severe inflammatory bowel disease (96, 97). While loss of IL-10 signaling affects Treg suppressive function on multiple cell types including T-cells leading to GI disease, there is evidence demonstrating that patients with IL-10R mutations fail to augment iTreg formation compared to healthy controls (112). Lastly, the immune regulatory role of perforin is demonstrated in patients with monoallelic perforin gene mutations resulting in partial degranulation defects that only present complications following an infection or other immune trigger such as cancer, whereby patients are unable to mount an appropriate immunological response (113). Thus, the role of perforin may be important for Treg mediated immune tolerance but may not be as critical as is the expression of other genes such as CTLA-4 and FOXP3 based upon the clinical consequences of loss-of-function defects.

Interestingly, the exact mechanisms used by CD4^+^ Treg to suppress remains incompletely understood, particularly *in vivo*. Treg dependency on different mechanisms *in vitro* and *in vivo* is likely attributed to the fact that different kinds of suppression might be necessary depending on the type, timing, intensity and duration of inflammation, and anatomical site of the immune reaction, as well as which cell needs to be suppressed i.e. T-cells, B cells, or APC. Here we review the role and importance of cytolysis as a mechanism of CD4^+^ Treg to module immune responses.

### CD4^+^ CyTreg Use Perforin Granzyme Pathway

An early study by Grossman et al. (2004) (18) demonstrated that following activation, human CD4^+^ tTreg expressed the tryptase GzA, whereas CD4^+^ pTreg expressed the serine protease GzB. Both CD4^+^ Treg subtypes were shown to kill autologous target cells *in vitro* in a perforin and CD18 dependent manner, suggesting that the interaction *via* the immunological synapse was necessary for cytotoxicity to occur (**Table 1**). They also demonstrated that activated T-cells and immature dendritic cells (DCs) were preferentially killed compared to resting T-cells and mature DCs, indicating that not all target cells have equivalent susceptibility to CD4^+^ Treg mediated killing (18). Interestingly, CD4^+^ tTreg had more potent *in vitro* killing compared to CD4^+^ pTreg (18). Differential granzyme expression and killing potential between Treg subsets suggest each subtype may have different roles in immune regulation. Notably, granzyme expression patterns also differ between different human T-cells subsets. While most resting NK cells and approximately half of CD8^+^ T-cells co-express GzA and GzB, very few resting CD4^+^ T-cells express both GzA and GzB (19). Unstimulated, freshly isolated human CD4^+^ Treg and CD4^+^CD25^neg^ T-cells express little to no GzB or perforin expression (18). Naive CD4^+^ T-cell activation with IL-2 alone leads to minor GzB expression, while activation with CD3/CD28 beads leads to intermediate percentage of GzB expressing cells with no GzA expression (19). The quantity of granzymes expressed based on level of activation in the different T-cell subsets supports the idea that some cells are more readily equipped to engage in cytolytic pathways. These data raise many questions that need to be further investigated such as: is the differential killing potential between CD4^+^ tTreg and pTreg associated with the differential granzyme expression? Why is it advantageous for human CD4^+^ tTreg to express GzA and CD4^+^ pTreg to express GzB?

Similar to human CD4^+^ Treg, freshly isolated murine CD4^+^ tTreg also have little to no GzB or perforin expression (80). The expression of these molecules is upregulated following activation with slower kinetics and quantity compared to that of CD8^+^ T-cells, and with greater quantity compared to that of CD4^+^CD25^neg^ T-cells (80). GzB deficient murine CD4^+^ tTreg showed reduced suppression of T-cell proliferation when compared to wildtype and perforin deficient CD4^+^ tTreg; suppression in this assay was due to increased T-cell apoptosis (**Table 1**) (81). It is unfortunate that GzA expression or its role in murine CD4^+^ tTreg killing was not evaluated in this particular study, as previous human CD4^+^ tTreg studies showed preferential expression for GzA (18). Furthermore, these results differ from the Grossman et al. (2004) study which showed human CD4^+^ Treg killing occurred in a perforin dependent mechanism; although notably the role of GzB was not evaluated (18). The mechanism by which GzB mediates cytolysis in the absence of perforin has yet to be fully described (81). It has been shown that GzB in the extracellular space can induce apoptosis of smooth muscle cells, offering an alternative pathway by which Treg can induce target cell death (114). Furthermore, it’s possible that Treg induced mechanical movement alone can induce necrosis of target cells, as killing in this form has been reported in the absence of perforin in T-cells (115). Altogether, these data demonstrate CD4^+^ Treg express killing molecules following activation and engage in cytolysis of immune cells through either a perforin or GzB dependent process. It is challenging to fully grasp the role perforin, GzB, and GzA in Treg killing, as each study typically measured the expression and tested the mechanistic role of only some of these molecules. For example, GzA is rarely investigated in Treg killing literature.

While it was originally known that CD4^+^ tTreg cells could directly suppress B cell proliferation, Ig production, and class switch recombination (116, 117), the exact mechanism used to suppress had not been described. Zhao et al. (2006) (80) demonstrated that activated murine CD4^+^CD25^+^ tTreg suppressed B cell proliferation in a cell contact-dependent, cytokine independent manner that was dependent on the upregulation of perforin and granzymes, and independent of the death receptors, Fas and TRAILR. They demonstrated that activated CD4^+^CD25^+^ tTreg preferentially killed antigen presenting B cells compared to resting bystander B cells in a GzB dependent, partially perforin dependent manner (**Table 1**) (80). They found freshly isolated CD4^+^CD25^+^ tTreg had little to no granzyme expression and could not engage in killing of B cells, but could potently suppress *in vitro* T-cell proliferation (80). In contrast, pre-activated CD4^+^CD25^+^ tTreg had increased expression of GzB and became licensed to kill B cells, but not T-cells (80). Why murine CD4^+^ tTreg in this study failed to kill T-cells is unclear as Gondek et al. (2005) (81) and Grossman et al. (2004) (18) reported mouse and human Treg could directly kill T-cells. A possible reason for the differences in these mouse studies might be that Treg in the Zhao et al. (2006) (80) study were stimulated with 5 µg/ml of plate bound anti-CD3 compared to 10 µg/ml of plate bound anti-CD3 in the Gondek et al. (2005) study (81). It’s possible that higher levels of activation were required to trigger sufficient perforin and granzyme molecule expression in order to stimulate T-cell killing. In summary, CD4^+^ tTreg use the perforin granzyme pathway to suppress B cell immune responses.

While CD4^+^ Treg had been reported to kill autologous immune cells, Choi et al. (2013) (98) was the first to explore whether CD4^+^ tTreg could co-opt this strategy to kill malignant cells. The idea that CD4^+^ Treg can be utilized to enhance anti-tumor immunity is paradigm shifting as Treg are associated with suppression of desired anti-tumor or anti-infectious responses. Choi et al. (2013) (98) demonstrated that an EGFRvIII-specific bispecific T-cell engager (BiTE) could redirect human CD4^+^CD25^+^CD127^dim/-^ Foxp3^+^ Treg and activate them in the presence of glioblastoma tumors expressing EGFRvIII. While human CD4^+^CD25^+^CD127^dim/-^ Foxp3^+^ Treg are known to be highly suppressive, these Treg showed increased expression of perforin, GzA, and GzB after activation (98). Furthermore, they potently lysed EGFRvIII^+^ tumor cells *in vitro* in the presence of EGFRvIII-specific BiTE and failed to lyse tumor in the presence of a non-specific BiTE, or when Treg were cultured in the absence of BiTEs. These human CD4^+^ tTreg killed EGFRvIII tumor cells in a perforin-dependent, partially GzB-dependent manner (98). Lastly, GzB^+^ Foxp3^+^ cells were identified in human primary glioblastoma tissues suggesting a potential role of cyTreg in the tumor microenvironment (TME) not previously recognized (98). Research will be necessary to determine which type of cyTreg, whether CD4^+^ pTreg or tTreg, would be more effective killers in the TME, and to determine what engages the cytolytic potential versus other suppressive mechanisms against tumor cells. Altogether, these data suggest that BiTEs, and potentially other therapies such as Chimeric Antigen Receptors (CAR), have the potential to redirect suppressive Treg to induce their cytolytic potential against tumor cells in an effort to promote anti-tumor responses. Whether CD4^+^ Treg redirected with BiTEs or CARs can engage in anti-tumor responses *in vivo* will need to be investigated.

Multiple groups had shown the role of the perforin and granzyme pathways in CD4^+^ cyTreg mediated suppression *in vitro*. However, it was unknown whether CD4^+^ cyTreg also regulated immune responses *in vivo*. Cao et al. (2007) (99) tested this idea using multiple tumor models and donor mice deficient in GzA, GzB, and perforin. They found that murine CD4^+^ Treg isolated from the TME upregulated GzB, but not GzA, and that perforin and GzB deficiency were essential in dampening anti-tumor responses *in vivo* (99). They further demonstrated in *ex vivo* experiments that murine CD4^+^ Treg derived from tumors killed NK and CD8 T-cells in a perforin, GzB dependent manner (**Table 1**) (99). This was the first report of CD4^+^ cyTreg using cytolysis similar to NK and CD8^+^ T-cells to suppress immune responses *in vivo*. Boissonnas et al. (2010) (82) further demonstrated the cytolytic potential of CD4^+^ cyTreg in anti-tumor responses using two-photon microscopy in explanted tumor draining lymph nodes (LN) to show that DC death only occurred when perforin sufficient Foxp3^+^ Treg were present and in the presence of tumor antigens (**Table 1**). A limitation of this study was the measured expression of both GzB and perforin in CD4^+^ Treg without mention of GzA, and the use of only perforin KO mice for *in vivo* studies. Thus, more research will be needed to understand GzA expression of CD4^+^ cyTreg in tumor models, determine the exact role of GzA and GzB for *in vivo* suppression, and evaluate CD4^+^ cyTreg anti-tumor potential *in vivo*. Altogether, these data suggest that CD4^+^ Treg utilize granzyme and perforin pathways to suppress anti-tumor responses *in vivo*.

To further support the role of cytolytic pathways in Treg suppression *in vivo*, Gondek et al. (2008) (83) demonstrated murine CD4^+^ tTreg initiated and maintained allograft tolerance in a GzB dependent, perforin independent manner. These results are in contrast with the two studies above which found Treg mediated killing was perforin dependent. Furthermore, gene expression analysis showed that mouse CD4^+^Foxp3^+^ iTreg expressed GzB, GzC, Fas-L, and DAPK2 (death-associated protein kinase 2) albeit at lower levels compared to CD8^+^ Foxp3^+^ iTreg. These results were supported through *in vivo* findings whereby CD4^+^ iTreg suppressed GVHD but abrogated GVL effects (8). Thus, the cytolytic potential of CD4^+^ iTreg compared to CD4^+^ tTreg *in vivo* will need to be evaluated. Lastly, Loebbermann et al. (2012) (84) sought to evaluate whether pulmonary responses were regulated by Treg during acute RSV infection in mice and found GzB deficiency in Treg worsened pulmonary pathology, suggesting GzB dependent suppression of lung inflammation during acute viral lung infection (**Table 1**). Unfortunately, they did not measure perforin or GzA expression of these Treg in the lungs or use KO mice to evaluate the role of these other killer molecules. In summary, these data suggest CD4^+^ Treg can use the perforin and/or granzyme pathway *in vitro* and *in vivo*, and have the potential to target DCs, B cells, T-cells, NK cells, and tumor cells to control immune responses (**Figure 1**).

### CD4^+^ cyTreg Protective Mechanims of GzB Induce Cell Death

Cytotoxic cells have mechanisms in place to prevent self-inflicting apoptosis from cytotoxic granule contents by expression of serine protease inhibitors called serpins. Serpin 6 (Spi6) has been demonstrated to protect murine Teff, DCs, and Treg from granzyme induced cytotoxicity (118–120). Similarly, the human equivalent of Spi6 is proteinase inhibitor 9 (PI9) and has been shown to be upregulated concurrently with GzB expression (121). Interestingly, Sula et al. (2017) (122) found that Treg from patients undergoing renal graft rejection, or Treg *in vitro* stimulated from healthy donors, had higher levels of GzB expression and higher GzB expression was shown to increase Treg apoptosis despite PI9 co-expression (122). Why PI9 was not protective of human Treg in this particular study will need to be further evaluated. Are these results due to differences between mouse and human Treg, or it may be possible that in these settings there was more GzB production than PI9 could neutralize? These data suggest that the granzyme-perforin pathway functions are a mechanism to suppress other target cells but also may serve as a mechanism for Treg activation induced cell death.

While we highlight studies that report killing as a mechanism of CD4^+^ Treg suppression, many Treg studies have found non-cytolytic mechanisms to be essential (78). A key factor that may help explain differences in regard to GzB expression and killing as a suppressive mechanism in Treg is whether rapamycin was used in Treg cultures to promote purity. Treg activated with anti-CD3, anti-CD28, IL-2 and rapamycin have lower levels of GzB expression when compared to Treg cultured in the absence of rapamycin; additionally, the lower expression of GzB was shown to be correlated with decreased levels of cytotoxicity (123). These results suggest that Treg cultured with rapamycin are likely to engage in other mechanisms of suppression outside the perforin-GzB pathway. Additionally, the measured killing by CD4^+^ Treg has been thought by some groups to be mediated by contaminating Teff. While the early studies used CD4^+^CD25^+^ and CD4^+^CD25^neg^ to differentiate Treg versus CD4^+^ T-cells, many noted key Treg characteristics such as constitutive CD25 expression and lack of IL-2 production or sorted out the top 2% of CD4^+^CD25^+^ T-cells to purposely gate out as many contaminating CD4^+^ T-cells (18, 100). Furthermore, in the Choi et al. (2013) (98) studies the Treg used for *in vitro* killing assays were first tested for *in vitro* suppressive function and were then used for *in vitro* killing assays with >95% Foxp3^+^. Thus, there is sufficient data to support CD4^+^ Treg can engage killing pathways in order suppress immune responses.

### CD4^+^ cyTreg Killing Pathways: FasL/Fas, TRAIL/TRAILR, Galectins

While the granzyme-perforin pathway appears to be important in CD4^+^ tTreg mediated control of immune responses, we must consider the role of other reported killing pathways. In an early report by Janssens et al. (2003) (100) murine CD4^+^CD25^+^ Treg were shown to be dependent on the Fas/FasL pathway to lyse APCs in an antigen-specific and MHC class II restricted manner (**Table 1**; **Figure 1**). These results showed that killing was a mechanism used by Treg to exert suppressive effects on APCs and bystander T-cells. Another mechanism of killing used by Treg is the tumor necrosis factor related apoptosis ligand (TRAIL)/death receptor 5 (DR5) pathway. Ren et al. (2007) (85) demonstrated that murine CD4^+^ Treg are dependent on the TRAIL/DR5 pathway to mediate both suppression and cytotoxicity *in vitro* and *in vivo* (**Table 1**). Using DR5 blocking antibodies, they showed CD4^+^ Treg used cytolysis to prolong tolerance to allogeneic skin grafts by killing CD4^+^ T-cells (**Figure 1**) (85). Lastly, whether CD4^+^ Treg use galectin-1 induced cell death to suppress effector T-cells *in vivo* will have to be further evaluated (124). Together, the current literature suggests that mouse and human CD4^+^ cyTreg predominantly engage in the perforin granzyme pathway, with some reported instances using the Fas/FasL and TRAIL/DR5 pathways. However, how CD4^+^ cyTreg choose one killing pathway versus another, and elucidating when they decide to engage in killing versus other suppressive mechanism warrants further investigation.

### Tr1

Type 1 regulatory (Tr1) cells are a T-cell subset characterized as Foxp3^neg^, CD49b^+^, and Lag3+ that produce high levels of IL-10 along with TGF-β, IFNγ, IL-5, and are IL-4^-^ and IL-2^low/-^ (125). While Tr1 cells are well recognized to suppress immune responses in cytokine dependent mechanisms *via* IL-10 and TGF-β (126, 127), Tr1 cells have also been found to engage in contact-dependent mechanisms including the PD1/PDL-1 and CTLA-4/CD80 pathways (128). Interestingly, Tr1 have also been found to kill myeloid cells through the perforin-granzyme pathway in an antigen dependent and independent manner (**Table 1**) (104, 129). Killing of APCs was shown to decrease T-cell activation and allow for bystander suppression. Furthermore, Grossman et al. (2004) (19) showed that human naive CD4^+^ T-cells stimulated with anti-CD3/CD46 antibody to generate Tr1 IL-10 producing cells led to expression of GzB in over 90% of the cells with no GzA expression, while anti-CD3/CD28 antibody mediated activation did not induce GzB expression. Based on this GzB expression pattern it was not surprising they found Tr1 cells showed maximal killing, whereas IL-2 activated CD4^+^ T-cells which had significantly lower levels of GzB expression showed minimal killing. These GzB^+^ Tr1 cells engage in perforin-dependent, MHC/TCR independent killing of allogeneic myeloid leukemia cell lines *in vitro* (**Table 1**) (19). In an effort to enhance Tr1 cell therapy, Roncarolo, who first identified Tr1 cells, and colleagues recently developed engineered human Tr1 cells by lentiviral transduction of IL-10 into peripheral CD4^+^ T-cells. This group found that these engineered human Tr1 cells had the ability to kill pediatric and adult acute myeloid leukemia (AML) cells (15, 130). Importantly, Tr1 cells retained their suppressive functions *in vivo* by suppressing GVHD and maintaining GVL responses (15). These data are exciting as it offers a cellular approach that is capable of both suppressing GVHD responses while concurrently potentiating GVL responses. How we can further enhance this type of bifunctional therapy will need to be evaluated as this approach can significantly address key limitations of Treg therapy for alloHSCT. In summary, Tr1 cells use the perforin granzyme pathway to suppress immune responses by targeting non-malignant myeloid cells and potentiate anti-tumor responses by killing malignant myeloid cells.

## The Role of CD8^+^ Treg in Immunosuppression and Cytolysis

CD8^+^ Treg represent another repository of cyTreg that remains substantially understudied. CD8^+^ Treg are loosely defined as a heterogenous population of CD8^+^ lymphocytes that can express several Treg associated surface markers and have immunosuppressive capacity, thus defining them as a Treg subset (131–133). CD8^+^ Treg have been reported to express a range of Treg markers such as CD122, CD25, CD103, GITR, CTLA-4, and PD1, and to engage in a range of cell contact-dependent and independent mechanisms to suppress immune responses (**Table 2**) (27, 134, 137, 138, 162–164). However, due to their low frequency *in vivo* and lack of conserved and consistent phenotypic markers, CD8^+^ Treg have yet to be fully described (163).

**Table 2:** Immunosuppressive and cytotoxic killing mechanisms used by CD8^+^ Treg subsets.

Treg Subtype	Source/Origin	Key Markers	Organism	Functional Mechanisms	References
CD8^+^CD122^+^	Thymus derived	CD122^+^ FoxP3^+/-^ PD-1^+^	Mouse	Fas/FasL dependent killing IL-10 mediated suppression	(134–136)
CD8^+^Foxp3^+^	*Ex vivo* induced (iTreg) Peripherally sourced (pTreg)	FoxP3^+^ CD25^+^ Lag3^+/-^ CTLA-4^+^ PD-1^+/-^ GITR^+/-^ CD28^+/-^ CD107a^+/-^	Mouse	+>- GzA/GzB dependent killing +>- perforin dependent killing Undefined contact-dependent suppression +>- CTLA-4 mediated suppression +<- IL-10 mediated suppression	(27, 63, 137, 138)
			Human	CCL4 mediated suppression +>- CTLA-4 mediated suppression +<- IL-35 mediated suppression +>- GzA/GzB dependent killing +>- perforin dependent killing	(27, 63, 139, 140)
CD8^+^CD103^+^	*Ex vivo* induced (iTreg) Peripherally sourced (pTreg)	CD103^+^ Foxp3^+/-^ CD25^neg^ CTLA-4^neg^ GITR^neg^ PD-1^neg^ IL-10^+^ TFG-β^+^	Mouse	Undefined contact-dependent suppression +/- GzA/GzB dependent killing	(141, 142)
			Human	Undefined contact-dependent suppression +/- GzA/GzB dependent killing	(27, 142, 143)
CD8αα^+^ IELs	Thymus derived	CD8αα^+^ CD8β^neg^ TCRαβ^+^ Foxp3^neg^ CD44^+^ CD69^+^ CD103^+^ Lag3^+^ CTLA-4^+^	Mouse	+/- IL-10 mediated suppression +/- Fas/FasL dependent killing +/- GzA/GzB dependent killing +/- TRAIL/DR5 dependent killing +/- Perforin dependent killing	(144–150)
	
CD4^+^CD8αα^+^ IELs	Peripherally sourced (pTreg)	CD4^+^CD8αα^+^	Mouse	IL-10 mediated suppression Perforin dependent killing	(151–153)
CD8^+^CD28^neg^	Peripherally sourced (pTreg)	CD28^neg^	Mouse	ILT3/ILT4 dependent killing	(154)
			Human	GzA/GzB dependent killing granulysin dependent killing ILT3/ILT4 dependent killing	(155–161)

GzB, granzyme B; GzA, granzyme A; ILT3/ILT4, immunoglobulin-like transcripts 3 and 4; CCL4, chemokine (C-C motif) ligands 4; IEL, Intraepithelial lymphocytes.

Symbols: +/- pathway or marker is shown to be intermittently applicable or inconsistently reported between multiple studies. +>- pathways is most often shown to be applicable. +<- pathway is most often shown to be unnecessary of cell function.

Like CD4^+^ Treg, CD8^+^ Treg are capable of inhibiting the activity of Teff. CD8^+^ Treg have been demonstrated to modulate Teff activation and proliferation through the release of immunosuppressive cytokines, including IL-10 and TGF-β (165), as well as inhibitory cell-to-cell interactions through CTLA-4 and PD-1 signaling pathways (166). Furthermore, the suppressive activity of CD8^+^ Treg *in vivo* has been shown to be important in regulating normal immune function and preventing inflammatory disease in humans, including inflammatory bowel disease, autoimmune diabetes, multiple sclerosis, and GVHD (167–169). In addition to their immunosuppressive capabilities, several subsets of CD8^+^ Treg have been described to utilize both suppressive and cytotoxic functions (CD8^+^ cyTreg), including both Foxp3^+^ and Foxp3^neg^ CD8^+^ Treg subsets described below. In fact, CD8^+^ cyTreg have been reported to utilize directed killing pathways as key mechanism to inhibit Teff activity (170). However, as the circulating frequency of CD8^+^ Treg is extremely low in both mouse and human (164), the vast majority of current literature focuses on different subsets of *ex vivo* generated CD8^+^ iTreg. As such, CD8^+^ cyTreg represent an extremely heterogenous population *in vivo*, and the precise mechanisms of suppression and/or killing utilized by these cell populations are highly dependent on the CD8^+^ cyTreg phenotype and local environmental stimuli (163, 171). The bifunctionality of CD8^+^ cyTreg remains highly debated in the literature and warrants further investigation.

### CCD8^+^Foxp3^+^Treg

CD4^+^ Treg are well defined by constitutive expression of Foxp3, the master regulator of Treg suppressive function (172–174). Similarly, both murine and human studies have also described Foxp3 expression in several subsets of CD8^+^ Treg, including CD8^+^CD25^+^Foxp3^+^, CD8^+^Foxp3^+^Lag3^+^, and CD8^+^CD103^+^Foxp3^+^ Treg (155, 175). In fact, several groups argue that Foxp3 expression in CD8^+^ T-cells is a highly conserved marker of CD8^+^ Treg (164, 176–178). CD8^+^Foxp3^+^ Treg have been shown to be highly immunosuppressive and in some circumstances have been shown to employ cytotoxic killing pathways as an additional mechanism of immunosuppression. In mice, CD8^+^CD25^+^Foxp3^+^ Treg have even been shown to be equally, if not more, suppressive *in vitro* than an equivalent CD4^+^ Treg (164). Therefore, CD8^+^Foxp3^+^ Treg have gained substantial interest as a unique cell type that may have applications in the treatment of some cancers, as well as autoimmune and inflammatory disease.

There are several subsets of CD8^+^ T-cells that have been reported to express Foxp3. CD8^+^CD25^+^Foxp3^+^ Treg are the most phenotypically and functionally similar to CD4^+^ Treg. Like their CD4^+^ counterpart, they express both CD25 and Foxp3 and have been found to co-express several additional Treg associated surface markers, including CTLA-4, Lag3, GITR and PD-1 (**Table 2**) (27, 179). However, CD8^+^Foxp3^+^ tTreg are present at extremely low levels in both human and mouse peripheral blood, ~0.4 and ~0.1%, respectively (164). This is significantly less than the frequency of circulating CD4^+^ Treg which constitute 1-3% of CD4^+^ lymphocytes in humans (180, 181) and ~5-15% in mice (164, 182). Mouse CD8^+^ Foxp3^+^ tTreg, with high CD25^+^ and GITR expression, were shown to suppress CD8^+^ T-cell responses in an influenza virus infectious model through an IL-10 dependent mechanism (137), whereas Cosmi et al. (2003) found that human CD8^+^CD25^+^Foxp3^+^ tTreg that expressed GITR and CTLA-4 could suppress the proliferation of autologous CD25^neg^ T-cells in a contact-dependent manner (138). To further support the importance of cytolytic pathways by Treg in dampening undesired immune responses, Correale and Villa (2008) found that CD8^+^ Treg from patients with multiple sclerosis could recognize and lyse myelin-specific CD4^+^ T-cells (183). Furthermore, they also found that lysis of these autoreactive T-cells was decreased when patients experienced exacerbations, and that killing occurred in a granule and MHC Class I dependent manner (183). Additionally, CD8^+^CD25^+^Foxp3^+^ Treg have been shown to have immunosuppressive properties in colorectal and prostate cancers with a potential to promote tumoral immune escape (178, 184).

Despite their low circulating frequency, several studies have demonstrated that human and murine CD8^+^Foxp3^+^ iTreg can be easily generated both *in vivo* and *ex vivo* (27, 63, 185). The generation of iTreg from CD8^+^CD25^neg^ Teff with robust antigen stimulation leads to the acquisition of Foxp3 expression and Treg associated immunosuppressive properties (27, 63, 185). Interestingly, the acquisition of immunosuppressive capabilities in CD8^+^CD25^+^Foxp3^+^ iTreg appears to coincide with the upregulation of cytotoxic molecules (27, 63). In addition to expressing CD25, Foxp3, CD28, CTLA-4 and GITR, CD8^+^CD25^+^ iTreg have also been shown to express high levels of cytotoxic molecules upon activation, including GzA, GzB and perforin in human CD8^+^ Treg and CD107α in both mice and human CD8^+^ Treg (**Table 2**) (27, 63). CD8^+^ cyTreg are proposed to utilize these killing pathways as another primary mechanism of suppression. However, the cytolytic potential of CD8^+^CD25^+^Foxp3^+^ iTreg remains highly debated in the literature, with one study reporting no observed *in vitro* killing capacity by allogeneic plasmacytoid dendritic cells induced human CD8^+^Foxp3^+^ iTreg, despite high expression of GzA and GzB (139) and others studies describing subsets of human CD8^+^CD25^+^Foxp3^+^ iTreg that simultaneously have both suppressive and cytolytic functions (27, 63).

Joosten et al. (2007) first described a subset of Lag3 expressing CD8^+^Foxp3^+^ Treg in both mice and humans that were shown to suppress human peripheral blood mononuclear cell (PBMC) proliferation through, at least in part, the secretion of CCL4 (chemokine (C-C motif) ligands 4) (63). CD8^+^Lag3^+^Foxp3^+^ Treg were shown to express CD107α, perforin and granulysin, and engage in directed killing in an antigen specific manner. Here, this study suggested that human CD8^+^ iTreg cytolytic function, but not suppression, is antigen dependent (63). They demonstrated that antigen primed CD8^+^Lag3^+^Foxp3^+^ Treg were able to kill infected but not uninfected macrophage targets, while the CD8^+^Lag3^+^Foxp3^+^ Treg were able to suppress Teff proliferation in a nonspecific manner. Expanding upon this earlier study, Mahic et al. (2008) described a subset of *ex vivo* induced human CD8^+^Foxp3^+^ iTreg that was also capable of both immunosuppressive and cytolytic functions (27). This subset of human CD8^+^Foxp3^+^ iTreg was shown to express high levels of perforin, GzA and GzB suggesting strong cytolytic potential. And although, several studies have reported CD8^+^ iTreg to secrete soluble factors such as IL-10, TGF-β, CCL4 and IL-35 (27, 63, 140, 163), transwell suppression assay analysis indicated that CD8^+^CD25^+^Foxp3^+^ iTreg rely on contact-dependent suppressive pathways (27). Several groups have suggested that CTLA-4 expression plays a major role in the contact-dependent suppressive function of CD8^+^CD25^+^Foxp3^+^ iTreg (16, 27, 139, 140, 163). However, the study by Mahic et al. (2008) demonstrated that contact-dependent CD8^+^ iTreg mediated suppression was maintained even in presence of CTLA-4, CD80 and CD86 blocking antibodies (27), suggesting that other suppressive mechanisms, such as cytolysis, may be at play in absence CTLA-4 mediated suppression.

### CD8^+^CD103^+^ Treg

Although Foxp3 expression in both CD4^+^ and CD8^+^ Treg has been shown to closely correlate with Treg suppressor function, there are now multiple reports that, unlike CD4^+^ Treg, the suppressive function of CD8^+^ Treg may not be dependent on the expression of Foxp3. In fact, several CD8^+^ Treg subsets, including CD8^+^CD103^+^ Treg and CD8^+^CD122^+^ tTreg, have been reported to be either Foxp3^neg^ or have only sporadic expression of Foxp3, while still maintaining immunosuppressive function (186, 187). Among these CD8^+^Foxp3^+/-^ Treg subsets, CD103 expressing CD8^+^ Treg are amongst the most investigated. In mice, CD103 is expressed by ~80% of CD8^+^CD25^+^Foxp3^+^ Treg (164), and can also be expressed by CD4^+^CD25^+^Foxp3^+^ Treg and CD8^+^Foxp3^neg^ Treg (188–190).

CD103 expression is a critical homing antigen for T-cells and assists in cell infiltration and residency in peripheral tissues (191, 192). The increased accumulation and persistence of T-cells in peripheral tissues is critical to maintain normal immune function. After CD8^+^ T-cells migrate into the periphery, CD103 expression is induced *via* a TGF-β signaling pathway (141, 193, 194). The induction of CD103 expression in CD8+ Teff is polyclonal and can lead to the development of an alloantigen-induced CD8^+^CD103^+^FoxP3^+/-^ Treg that possess immunosuppressive capabilities, regardless of Foxp3 status (143, 188). Although CD103^+^ Treg have been shown to produce both IL-10 and TGF-β, a majority of the current literature suggests that the immunosuppressive function of human CD8^+^CD103^+^FoxP3^+/-^ Treg is contact-dependent and does not rely on the production of soluble factors (**Table 2**) (142, 143). Although, it has also been reported that human CD8^+^CD103^+^Foxp3^neg^ Treg did not express to PD-1, GITR and CTLA-4 (142), suggesting that CD8^+^CD103^+^Foxp3^neg^ iTreg must be employing different mechanisms of suppression compared to other CD8^+^ Treg subsets and that CD8^+^CD103^+^Foxp3^neg^ Treg may rely on cytolytic killing mechanisms to suppress T-cell activity. However, despite several studies suggesting that human CD8^+^CD103^+^ iTreg may retain their cytolytic capacity and engage in directed killing of Teff following antigen stimulation (27, 142), a majority of the current literature suggests that CD103 expression is not a conserved marker of CD8^+^ Treg cytolytic activity in mice or humans (142, 143, 188, 195). Further, many of these studies suggest that, unlike other CD8^+^ Treg subsets, a majority of CD8^+^CD103^+^Foxp3^neg^ Treg suppress Teff through non-cytotoxic mechanisms and have very little cytolytic function (142, 143, 188, 195). While these reports do indicate that some CD8^+^CD103^+^ Treg may have both suppressive and cytolytic potential, the current the literature is still unable to phenotypically distinguish between the immunosuppressive, cytolytic and dual function populations. A possible explanation to these varying mechanisms used by CD8^+^CD103^+^ Treg is that CD103 expression is likely not a conserved marker of CD8^+^ Treg, as several studies have also reported CD103 expression as a marker of activated tissue-resident memory T-cells (Trm) (196, 197). In fact, CD8^+^CD103^+^ Trm cells have been shown to be significant contributors to anti-tumor immunity due to their substantial cytotoxicity and cytokine production potential (196–199). Despite this, CD8^+^CD103^+^ cyTreg are an increasingly interesting Treg population that necessitates further investigation.

### CD8^+^CD103^+^Foxp3^neg^ Treg

Thymus derived CD8^+^CD122^+^Foxp3^neg^ T-cells represent a subset of T-cells that can suppress autoimmunity, anti-tumor responses, and allogeneic responses (200–202). While CD122 expression in T-cells is often associated with CD8^+^ T stem memory (Tsm) populations (203–205), murine CD8^+^CD122^+^ T-cells have also be shown to be potent suppressors of allograft rejection (162). Murine CD8^+^CD122^+^Foxp3^neg^ Treg have been reported to recognize activated T-cells *via* MHC class I/TCR and suppress T-cell activity *via* IL-10 production (134). It has been shown that the PD1 expression in murine CD8^+^CD122^+^ T-cells was critical for the enhanced suppressive function and that IL-10 was partially responsible for the suppression of allograft rejection (134). A follow up study demonstrated that murine CD8^+^CD122^+^PD1^+^ T-cells suppressed Teff proliferation *in vitro* in an IL-10 dependent manner and could also kill Teff in a Fas/FasL dependent manner (135). The use of cytolysis as a mechanism of CD8^+^CD122^+^ T-cells to modulate immune responses was further supported in a skin allograft model where deficiency of FasL expression, or inhibition of this pathway with blocking antibodies, abrogated suppression of allograft rejection (135). Furthermore, Akane et al. (2016) reported that murine CD8^+^CD122^+^ T-cells, particularly the CD49b low expressing CD8^+^CD122^+^ T-cells, were capable of suppressing activated CD4 and CD8 T-cells in a Fas/FasL dependent manner, and in an MHC class I/TCR dependent process (136). Together, these data emphasize the importance of CD8^+^CD122^+^ T-cells as an immunoregulatory cell type that prefers IL-10 and Fas/FasL pathways to suppress immune responses.

### CD8aa^+^ Intraepithelial Lymphocytes

Intraepithelial lymphocytes (IELs) are a predominant T-cell population strategically dispersed in the intestinal epithelial layer where they contribute as a first line of defense against infections to protect the mucosal barrier (151, 206–208). IELs can be divided into two main categories: induced IELs, which are conventional CD4^+^ or CD8αβ^+^ T-cells that have undergone extrathymic differentiation in the intestines (151), and natural IELs, which express CD8αα with TCRαβ^+^ or TCRγδ^+^ and have been well-documented to develop in the thymus before migrating to the gastrointestinal tract (207–209).

Induced CD4^+^CD8αα^+^ IELs, or CD4 T-cells that have peripherally acquired CD8αα^+^ through the Th2 lineage pathway, are significantly detected under heightened immune responses (151). CD4^+^CD8αα^+^ IELs are producers of Treg-associated cytokines IL-10 and TGF-β, and can suppress Th1-induced intestinal inflammation in an IL-10-dependent manner to protect the mucosal barrier (151). CD4^+^CD8αα^+^ IELs also exhibit cytolytic activity through perforin expression (152). However, upon significant pathogenic infiltrations, activated CD4^+^CD8αα^+^ IELs can contribute to the pathological progression of inflammatory bowel disease through release of proinflammatory cytokines TNF-α, IL-15, and IFN-γ with upregulation of CD107a (152). IL-10 has been shown to suppress infiltration of gluten-dependent cytolytic CD4^+^CD8αα^+^ IELs for potential prevention of celiac disease (153).

Antigen-experienced natural CD8αα^+^CD8β-TCRαβ^+^ IELs (CD8αα^+^ IELs) amount to nearly 40% of the T-cell population within the intestinal layer and are of considerable interest due to their potential for dual immunosuppressive and cytolytic functions (206). CD8αα^+^ IELs express the activation markers CD44 and CD69 from thymic development in the presence of high affinity self-antigen agonists (207, 208, 210). Though self-reactive, CD8αα^+^ IELs are not self-destructive (144) and maintain a regulatory role within the gut, constitutively expressing CD103 (208) and highly expressing Lag3, CTLA-4 (145), and NK associated genes (145, 208, 211) including the inhibitory Ly49 receptors, CD16, CD122, and NK1.1, but with very low expression of Foxp3 mRNA (145). In the absence of their specific MHC-restricted antigen, these cells were found to be enriched for TGF-β, IL-10 and IFNγ mRNA, suggesting that these cells either constitutively express these immunoregulatory cytokines or express them through non-TCR-mediated signals (144, 145). However, upon activation, CD8αα^+^ IELs substantially reduce mRNA expression of these cytokines (144, 145). In slight contradiction, another study could not detect IL-10 secretion or IL-10R expression in either *in vitro* non-activated or anti-CD3/CD28-activated CD8αα^+^ IELs (146). However, adoptive transfer of CD8αα^+^ IELs into SCID mice did prevent CD4^+^ T-cell-induced colitis in an IL-10-dependent manner; CD8αα^+^ IELs derived from IL-10 knockout transgenic mice were ineffective for disease prevention. It has been proposed that murine gastrointestinal epithelial cells, which constitutively express IL-10R, rely on IL-10-dependent signals from CD8αα^+^ IELs (146).

The cytotoxic potential of CD8αα^+^ IELs has also been debated. A study in which CD8αα^+^ IELs with LCMV-reactive TCRs were activated by LCMV infection, no cytotoxic activity could be detected (144), albeit several other studies have confirmed expression of perforin, GzA, GzB, and FasL (147, 148). *In vivo* wild-type CD8αα^+^ IELs constitutively express GzB but not GzC (147, 148); GzB knockout CD8αα^+^ IELs were observed to upregulate granzyme C for non-redundant protection in a murine model of cytomegalovirus (CMV) infection (147). Reovirus 1/L-stimulated IELs were shown to effectively utilize Fas/FasL, perforin, and TRAIL-mediated cytotoxicity pathways (149, 150). As such, CD4^+^CD8αα^+^ and CD8αα^+^ IELs are an enigmatic “activated yet resting” cell population that maintains immunoregulatory and cytolytic functions in mucosal tissues.

### CD8^+^CD28^neg^ Treg

CD8^+^CD28^neg^ T-cells have also been reported to exhibit both cytotoxic and immunosuppressive function. However, not unlike most other CD8^+^ Treg subsets, the current literature describes CD8^+^CD28^neg^ T-cells that are either immunosuppressive or cytolytic. While several studies have reported human CD8^+^CD28^neg^ T-cells possess high cytotoxic potential due to high expression of cytolytic molecules, including perforin, GzA, GzB and granulysin (156–160), other studies have reported a subset of CD8^+^CD28^neg^ T-cells with distinct lack of cytotoxic function, but capable of immunosuppressive function (154, 155, 161). Interestingly, CD8^+^CD28^neg^ Treg have been suggested to induce a unique contact-dependent suppressive pathway to inhibit alloreactive Teff. Several early studies demonstrated that human and mouse CD8^+^CD28^neg^ Treg are able to promote the tolerization of APCs by both inducing the upregulation of immunoglobulin-like transcripts (ILT), ILT3 and ILT4, and simultaneously downregulation of costimulatory molecule expression on APCs. This in turn impaired APC/CD4^+^ T-cell interactions, reduced IFNγ production, and suppressed the activity of alloreactive T-cells (154, 155, 161). Despite these early reports of immunosuppressive function, there remains no comprehensive phenotypic definition of the immunosuppressive CD8^+^CD28^neg^ Treg population and efforts towards an accurate and comprehensive functional description of CD8^+^CD28^neg^ Treg have also been impeded by the failure of current studies to identify conserved surface markers to distinguish between the immunosuppressive and cytotoxic subpopulations without functional analysis. Despite this, CD8^+^CD28^neg^ Treg do offer an interesting avenue for further study as *in vivo* studies have highlighted their important role in immune regulation and may offer a novel approach to Treg-based immune therapies (154, 161).

### CD8^+^Ly49/KIR^+^Foxp3^neg^ Treg

Another CD8^+^ Treg subset important for immune regulation expresses either Ly49 or killer immunoglobulin receptors (KIRs) in mouse and human CD8^+^ T cells, respectively. Early studies demonstrated that CD8^+^CD44^+^ICOSL^+^Foxp3^neg^ Treg recognized the Qa-1/peptide complex on T follicular helper cells (T_FH_) to promote tolerance to self *via* the perforin pathway (212). Using a Qa-1 knock in mouse model that impaired CD8^+^CD44^+^ICOSL^+^Foxp3^neg^ Treg activity, it was shown that mice developed a lupus-like autoimmune disorder that was associated with T_FH_ cell dysregulation, increased autoantibodies, and severe glomerulonephritis (212). It was then shown that these CD8^+^CD44^+^ICOSL^+^Foxp3^neg^ Treg subset had high expression of CD122 and uniquely expressed Ly49 (213). Ly49 is a member of a family of C-type lectin receptors that can be expressed on NKT cells, IELs, macrophages, DCs and a fraction of CD8+ T cells. However, Ly49 is ubiquitously expressed on NK cells, enabling those cells to distinguish between healthy, infected, or altered cells (214). CD122 expression in combination with Ly49 on NK cells may explain its dependency on IL-15 for development and function (212). Furthermore, a group found that B6-*Yaa* mice, which also develop a lupus-like autoimmune disorder that is exacerbated with IL-15 receptor deficiency (215), have increased numbers of T_FH_ and germinal center B cells with defective CD8^+^ Treg suppressive function. These data suggest a role of CD8^+^CD122^+^CD44^+^Ly49^+^Foxp3^neg^ Treg in B6-*Yass* mice lupus-like pathogenesis (213). To further extend our understanding of CD8^+^Ly49^+^ Foxp3^neg^ Treg in autoimmunity, a group demonstrated that clonally expanded CD8^+^Ly49^+^ T cells in a model of experimental autoimmune encephalitis (EAE) represent a CD8^+^Ly49^+^Foxp3^neg^ Treg subset that is non-responsive to myelin protein but is instead suppressive towards autoreactive CD4^+^ T cells. The suppressive mechanism of this CD8^+^Ly49^+^Foxp3^neg^ Treg was found to occur in a perforin dependent manner, similar to previous reports of this pathway required by CD8^+^ Treg to suppress T_FH_ cells (212, 216). A recent report confirmed the existence of a population equivalent to CD8^+^Ly49^+^Foxp3^neg^ Treg suppressive subset in humans. Since Ly49 genes are not present in the human genome, human CD8^+^ Treg were found to express the killer immunoglobulin-like receptors (KIRs) which have parallel functions (217, 218). Human CD8^+^KIR^+^Foxp3^neg^ Treg are increased in patients with autoimmunity or infection as compared to healthy counterparts (219). Using *in vitro* assays, it was demonstrated that CD8^+^KIR^+^Foxp3^neg^ Treg suppressed gliadin specific CD4^+^ T cells isolated from patients with celiac disease. They found that suppression by human CD8^+^KIR^+^Foxp3^neg^ Treg occurred in a contact dependent manner, associated with increased annexin V expression in pathogenic gliadin specific CD4^+^ T cells, consistent with murine studies of perforin dependent suppression by CD8^+^Ly49^+^Foxp3^neg^ Treg (219).

## Role and Applications for cytotoxic Treg in the Treatment of GVHD

CyTreg may offer a novel approach to the treatment of GVHD that is underrepresented in current clinical research. One of the biggest hurdles to the development of a successful GVHD therapy is the preservation of the therapeutic GVL effect. With current CD4^+^ Treg based therapies for GVHD there is a risk of suppressing GVL activity, resulting in relapse in alloHSCT recipients (8). Remarkably, the dual immunosuppressive and cytotoxic action of cyTreg has been shown in pre-clinical studies to alleviate acute GVHD (aGVHD) while preserving the essential GVL activity of the graft (8, 15, 16). For example, CD4^+^ Tr1 cells have been shown to both suppress GVHD and preserve GVL responses *in vivo* (15). There is also evidence supporting a role of CD4^+^ cyTreg in the alloHSCT setting. A group investigated which killing mechanisms were necessary for CD4^+^ tTreg suppression of anti-tumor responses in the allogeneic setting by using RMAS lymphoma and B16 melanoma cells derived from C57BL/6 mice and injected them into 129/SvJ mice to create a minor histocompatibility mismatch (99). Using GzB KO mice, they demonstrated that CD4^+^ tTreg used the GzB pathway to non-redundantly suppress anti-tumor responses *in vivo* (99). They then posited whether tTreg would also be dependent on GzB to suppress GVHD following alloHSCT in a murine major histocompatibility mismatch model. Similar to their original tumor studies, they found that murine tTreg upregulated GzB expression in the allosetting (220). However, in contrast to their tumor studies they found that GzB was non-essential for GVHD mediated suppression, as mice treated with wildtype and GzB KO CD4^+^ tTreg had comparable survival curves, decrease in serum cytokines, and protection of aGVHD target organs (220). Interestingly, another group found that the hypomethylating agent azacytidine could be used to enhance CD4^+^Foxp3^+^ Treg induction following alloHSCT and that murine aGVHD suppression *via* these CD4^+^ iTreg occurred in a GzB independent, and partially perforin dependent manner without abrogating the GVL response (101). It is unclear why aGVHD suppression was GzB independent in this latter two studies; however, it is plausible that the different source of Treg used for aGVHD suppression may have a different transcriptome and thus be dependent on different pathways to suppress similar allosettings. Overall, these studies demonstrate that both CD4^+^ tTreg and iTreg can use the perforin-granzyme pathway to dampen alloimmune responses *in vivo*, and that cyTreg have the capacity to suppress aGVHD while maintaining anti-tumor responses in most circumstances.

Furthermore, several studies have demonstrated an integral role for CD8^+^ iTreg in aGVHD pathology. Zheng et al. (2013) (16) demonstrated that *ex vivo* human CD8^+^CD25^+^Foxp3^+^ iTreg were capable of controlling GVHD while preserving the GVL effect. Here, human CD8^+^ iTreg GVHD suppression was mediated through a CTLA-4 dependent mechanism, which resulted in reduced T-cell proliferation and production of inflammatory cytokines in target organ systems resulting in improved GVHD outcomes (16). Similarly, Heinrichs et al. (2016) (8) demonstrated that combinational therapy using both mouse CD8^+^ and CD4^+^ iTreg, but not CD4^+^ iTreg alone, was capable of suppressing aGVHD while maintaining GVL responses in mice. These results suggest that CD8^+^ iTreg can play an integral role in the maintenance of GVL activity.

Interestingly, it has been shown recently that CD8^+^Foxp3^+^ iTreg alone are sufficient to prevent aGVHD, even in absence of CD4^+^ iTreg. Beres et al. (2012) (17) demonstrated that the adoptive transfer of human CD8^+^Foxp3^+^ iTreg into humanized recipient mice, which lack the ability to make both murine CD8^+^ and CD4^+^ iTreg (Rag2^−/−^gc^−/−^), significantly ameliorated the severity of aGVHD, protected recipient mice from death and preserved the GVL response. Not only did this study support the protective role of CD8^+^ iTreg against aGVHD while preserving GVL activity, but it also showed that CD8^+^ pTreg are induced *in vivo* early post-transplant (17). These data suggest that CD8 iTreg and pTreg may play a significant role in the regulation of inflammation during the early phases of aGVHD and support that notion CD8^+^Foxp3^+^ iTreg offer an approach for the GVHD suppression and GVL maintenance. The potential applications for a dual purpose GVHD/GVL Treg-based cellular therapy is not limited to aGVHD. CD8^+^CD103^+^ iTreg have also been shown to alleviate chronic GVHD (cGVHD). In a mouse model of cGVHD with lupus syndrome, Zhong et al. (2018) (46) demonstrated that the adoptive transfer of murine CD8^+^CD103^+^ iTreg ameliorated cGVHD severity and enhanced survival. They observed a significant reduction in autoantibodies and renal injury, in conjunction with reduced Th and B cell responses. These data were supported by a follow-up study that also demonstrated the therapeutic effect of murine CD8^+^CD103^+^ iTreg adoptive transfer in a mouse model of cGVHD and lupus nephritis (221). They demonstrated that the immunosuppressive function of CD8^+^CD103^+^ iTreg was closely associated with expression of CD39, the rate limiting enzyme in the production of immune suppressive adenosine (221).

Human CD8^+^CD103^+^ iTreg have also been demonstrated extremely stable under inflammatory conditions *in vivo* and play a critical role in preventing kidney injury in patients (222). As such, CD8^+^CD103^+^ iTreg adoptive transfer provides a novel approach for the treatment of kidney disease as well as other autoimmune and inflammatory diseases (158, 221). However, El-Asady et al. reported that CD103 expressing mouse CD8^+^ T-cells may have the potential to exacerbate aGVHD (141). They demonstrated that host derived CD8^+^ Teff that migrate to the intestinal epithelium can also gain CD103 expression *via* TGF-β signaling. The resulting population has an enhanced capacity to accumulate within the gut tissue resulting in a concentration of activated CD8^+^ Teff in the gut tissue that exacerbated host intestinal injury (141). This suggests that a subset of CD8^+^CD103^+^ T-cells may not have immunosuppressive activity as reported by other studies (46, 142, 143, 188, 221). This again aligns with the notion that our current understanding of CD8^+^ cyTreg populations is greatly limited by the lack of conserved surface markers which would help differentiate cyTreg *vs.* Teff. In spite of this, these early studies provide encouraging data that suggests select subsets of CD4^+^ and CD8^+^ cyTreg, either alone or in combination, may provide a novel approach to suppress GVHD and maintain GVL responses.

## Car-Treg: Could Cytotoxic Car Treg Offer a New Therapeutic Avenue?

The generation of antigen specific Treg is of particular interest because of their increased potency compared to polyclonal Treg and their potential to decrease the risk of non-specific immunosuppression (223). The remarkable success of CAR T-cell therapy to induce remission in relapsed and/or refractory hematological malignancies has warranted their application in other cell types and disease models (224, 225). CARs are synthetic receptors that consist of an extracellular single chain variable fragment (scFv) linked *via* a hinge and transmembrane domain with an intracellular CD3 activation domain and depending on the CAR generation typically contain 0-2 costimulatory domains. CARs can be advantageous compared to T-cell receptor (TCR) guided approaches when T-cells are unable to sufficiently recognize and activate in response to antigen in an MHC restricted manner (226). CARs have been used to redirect Treg to target 2,4,6-trinitrophenol (TNP), carcinoembryogenic antigen (CEA), factor VIII (FVIII), myelin oligodendrocyte glycoprotein (MOG), human leukocyte antigen A2 (HLA-A2), and CD19 in preclinical models of colitis, hemophilia, multiple sclerosis, and transplantation, respectively (224, 227). Despite reports of cytotoxicity as a mechanism of suppression by Treg (77), it is surprising that the majority of the CAR Treg studies published to date have found negligible to minimal cytotoxicity towards antigen expressing target cells (228–232). Lack of cytotoxicity has been beneficial for tissue-specific CAR Treg generated to protect the target tissue. However, the lack of cytotoxicity by these CAR Treg could be due to the experimental conditions used to generate CAR Treg or measure killing.

Some groups have recently targeted B cells using CD19 specific CAR (CAR19) Treg in xenogeneic models of skin transplantation and GVHD. Imura et al. (2020) found minimal to negligible *in vitro* killing by human CAR19 Treg (233). While CD19 CAR Treg engaged in negligible killing of CD19 target cells using a 1:1 E:T ratio, a higher E:T ratio demonstrated CAR19 Treg could kill 17% of CD19 target cells compared to 60% killing mediated by CAR19 Teff. Boroughs et al. (2019) reported human CAR19 Treg killed approximately 45% CD19 B cells *in vitro* at a 1:1 ratio using the perforin-granzyme pathway (234). Imura et al. (2020) (233) argued that their negligible CAR19 Treg killing was associated with a higher Treg purity as they used CD4^+^CD25^hi^CD127^lo^CD45RA^+^ Treg, whereas Boroughs et al. (234) used bulk CD4^+^CD25^hi^CD127^lo^ Treg which contained CD45RO^+^ cells that have been shown to behave more like effectors based on lower expression of Treg markers and higher production of pro-inflammatory cytokines (233). However, Boroughs et al. (234) directly tested this hypothesis and found that CD45RA^+^ CAR Treg displayed equal killing when compared to bulk Treg. They also generated an EGFR specific CAR Treg in the same conditions as CD19 CAR Treg and demonstrated it had minimal but measurable killing of antigen EGFR^+^ skin grafts (234). Koristka et al. (235) also found that their human UniCAR-CD28 Treg killed approximately 20% of target cells, whereas the 4-1BB based CAR Treg killed about 10% of targets. Together, these studies demonstrate CAR Treg have the potential to engage in killing of target cells *in vitro* and *in vivo*.

The CAR design, antigen targeted, the affinity or signal strength of the CAR, whether CARs undergo tonic signaling, CAR mediated exhaustion, or cell intrinsic mechanisms of Treg may all influence cytotoxicity. The majority of CAR Treg studies that compared the CD28 and 4-1BB costimulatory domains found that CD28 costimulation was superior based on increased expression of Foxp3, CTLA-4, and Helios, IL-10 production, and enhanced suppressive function both *in vitro* and *in vivo* (233, 234, 236). Another group reported that human CAR Treg with the 4-1BB costimulatory domain produced less inflammatory cytokines and were less cytolytic compared to the CD28 domain, suggesting CAR Treg with 4-1BB domain may be more stable *in vivo* (235). In contrast, Boroughs et al. (2019) (234) found that human CAR19 Treg with either 4-1BB or CD28 costimulatory domains had comparable *in vitro* killing, suggesting killing was not dependent on the costimulatory domain used. Further research will be necessary to evaluate optimal CAR Treg design and determine whether a specific costimulatory domain has the potential to reduce or enhance Treg cytotoxicity. Secondly, whether antigen specificity or affinity may have a role in the induction of cytolytic mechanisms in CAR Treg remains to be determined. Boroughs et al. (234) evaluated whether higher scFv affinity played a role in cytolytic induction of CAR Treg. To do this, they generated a CAR with the same components as the original construct except now the scFv was directed to EGFRvIII which has been reported to have a significantly lower affinity to its antigen compared to the CD19 scFv (EC50 of ~100 ng v 6 ng). Using EGFRvIII^+^ target cells, they found that EGFRvIII specific CAR Treg could induce comparable target lysis to that measured by CD19 CAR Treg killing (234). These data suggests the affinity of the CAR does not play a role in the cytolytic potential Treg. Lastly, the first human CD8^+^ CAR Treg study found that CD8^+^ anti-HLA-A2 CAR Treg with the CD28 costimulatory domain had no cytotoxicity activity toward HLA-A2 kidney endothelial cells (229). These results align with other anti-HLA CAR Treg studies and are interesting as CD8^+^ Treg are thought to be more cytolytic than CD4^+^ Treg. How to co-opt, or prevent, the cytolytic potential of Treg will need to be determined to ensure safety of CAR Treg therapy.

CD19 CAR CD8^+^ T-cells can suppress B cell mediated autoimmune disease by killing B cells, at the expense of significant risk for cytokine release syndrome (CRS) (237–239). CAR Treg have the potential to engage in cytolytic mechanisms without CRS due to their immunosuppressive potential and relative lack in proinflammatory cytokine production. Such cytolytic CAR Treg could be evaluated in autoimmune disease and in transplantation of recipients with B cell mediated hematologic malignancies. B-cell specific CAR Treg may have the potential to kill pathogenic B-cells and induce bystander suppression to dampen deleterious and excessive inflammation associated with autoimmunity and GVHD (231, 240). It has been reported that non-cytolytic, human CD19 CAR CD4^+^ Treg compared to human CD19 CAR CD8^+^ T-cells maintain weights and clinical scores in models of GVHD with no measurable increase in IL-6 production, one of the CRS hallmarks, suggesting CAR Treg may have a lower risk for CRS (233); whether cytolytic CAR19 Treg also prevent CRS will need to be investigated. Cytolytic CAR Treg may offer a new therapeutic approach that allows for suppression of excessive, pathologic inflammatory responses while simultaneously inducing apoptosis of B-cells that produce pathogenic antibodies as well as present antigen to potentiate disease. Unfortunately, B-cell specific CAR Treg therapy would also target non-pathogenic B-cells leading to B-cell aplasia. Suicide genes, cell surface antigens that can be targeted by antibodies, cytolytic function induced upon activation, or logic gate to control cell decisions to kill or spare a given cell population may be necessary to regulate cytolytic CAR Treg function *in vivo* (241). In conclusion, HLA-A2 CAR Treg demonstrate minimal to negligible cytotoxicity, while CD19 CAR Treg studies show measurable *in vitro* cytotoxicity. These data support a potential role of for CD19 CAR Treg to engage in cytotoxicity *in vivo*. Thus, there is dire need to study whether cytotoxicity is present in various CAR Treg constructs, as well as understand how it’s regulated so we can ensure safety and efficacy.

## Conclusion and Future Directions

Our understanding of Treg biology has significantly improved over the last 20 years. However, much is left to understand how Treg behave *in vivo* and what mechanisms are required for their effective control of immune responses. Mounting evidence demonstrates that there are multiple subtypes of regulatory T-cells within CD4^+^ T-cells and CD8^+^ T-cells, and some which do not require Foxp3 expression. A highly debated and controversial topic has been cytolysis as a mechanism of suppression by Treg. Some have argued that the measured killing by Treg is explained by impurities of Treg culture and attributed to contaminating Teff. However, others have directly addressed these concerns by sorting pure populations of Treg and demonstrated comparable Treg killing. Mouse and human Treg studies support cyTreg as a suppressive regulatory cell capable of dampening inflammatory immune responses *in vivo*, as well as capable of utilizing cytolytic mechanisms towards target cells in order to regulate immune responses. There are multiple reports of CD4^+^ and CD8^+^ cyTreg, as well as Tr1 cells engaging in killing mechanisms to effectively suppressing an inflammatory milieu, such as GVHD, while maintaining or possibility potentiating killing responses, such as GVL. There is evidence to support that CD8^+^ Treg are equally, if not more, suppressive *in vitro* than an equivalent CD4^+^ Treg. Although CD8^+^ Treg may not be better suppressors when compared to CD4^+^ Treg in GVHD studies they do offer the key advantage of potently maintaining the GVL response. Further, when CD4^+^ and CD8^+^ iTreg are combined, GVHD suppression with maintenance of the GVL effect are improved as compared to either subset alone. Additionally, it appears that while CD8^+^Foxp3^neg^ Treg do play a role in immune suppression they are not sufficient to solely maintain immune homeostasis and tolerance, as IPEX patients with Foxp3 mutations in both CD4^+^ and CD8^+^ Foxp3^+^ Treg populations experience severe immune dysregulation. CD4^+^Foxp3^+^ Treg are equally, or more potent, in immune regulation than CD8^+^Foxp3^+^ Treg, although in certain situations, CD8^+^Foxp3^+^ would be the more desirable population. Thus, continued investigations as to the optimal regulatory subtypes will be critical to enhance Treg cell therapy for various disease models, particularly for transplantation and autoimmune disorders.

cyTreg offer a new avenue for Treg cell therapies. cyTreg would be highly beneficial in the context of alloHSCT whereby GVHD suppression and GVL maintenance could be both achieved. It would also be of interest in B-cell mediated diseases (e.g. autoimmune disorders) whereby suppression of highly inflammatory environments is necessary and killing of the pathogenic B-cells would suppress autoimmune responses. Furthermore, cyTreg would be highly advantageous in chronic infection models to dampen excessive inflammation and where killing of infected cells would be desired. However, cyTreg could be highly detrimental in the setting of CAR Treg redirected to alloantigens to suppress solid organ allografts. With Treg cell therapies currently under investigation in early clinical trials for solid organ transplant, alloHSCT, and autoimmune diseases, it will be imperative to explore the potential cytotoxicity of these therapies. Due to low frequencies of CD4^+^ Treg and CD8^+^ Treg, genetic engineering of potent cyTreg or improved methods for either *in vitro* or *in vivo* induction, expansion or activation will be necessary to increase the therapeutic index of these Treg cell therapies. The lack of a comprehensive phenotypic and functional definition of CD8^+^ cyTreg subtypes and CD8^+^ Teff populations continues to hinder the development of cyTreg based bifunctional therapies for clinical translation. As such, further strides are necessary to clearly distinguish between Teff and cyTreg populations. Thus, there is a critical need to investigate what mechanisms regulate cyTreg cytotoxicity in an effort to develop and optimize Treg cell therapies for each disease model and disease. Altogether, cyTreg offer an exciting avenue to expand our understanding of Treg biology, as well as develop safer and more effective Treg therapies for clinical use.

## Author Contributions

SBW and JHL contributed equally to the writing and editing of this manuscript, as well as table generation. SJ contributed with writing, editing, and making of the figure. BRB contributed with writing and editing of the manuscript. All authors contributed to the article and approved the submitted version.

## Funding

Authors were supported by funding from the National Institutes of Health. RO1 HL56067, R01 HL11879, R01 HL155114, R37 AI 34495, and P01 AI05662. T32 AI007313 and F30HL156312.

## Conflict of Interest

BRB receives remuneration as an advisor to Magenta Therapeutics and BlueRock Therapeutics; Research funding from BlueRock Therapeutics, Rheos Medicines, Carisma Therapeutics, Inc., and is a co-founder of Tmunity Therapeutics.

The remaining authors, SBW, JHL and SJ, declare that this article was written in the absence of any commercial or financial relationships that could be construed as a potential conflict of interest.

## Publisher’s Note

All claims expressed in this article are solely those of the authors and do not necessarily represent those of their affiliated organizations, or those of the publisher, the editors and the reviewers. Any product that may be evaluated in this article, or claim that may be made by its manufacturer, is not guaranteed or endorsed by the publisher.

## References

[1] Sakaguchi S Sakaguchi N Asano M Itoh M Toda M. Immunologic Self-Tolerance Maintained by Activated T Cells Expressing IL-2 Receptor Alpha-Chains (CD25). Breakdown of a Single Mechanism of Self-Tolerance Causes Various Autoimmune Diseases. J Immunol (1995) 155(3):1151–64. 7636184

[2] Taylor PA Noelle RJ Blazar BR. CD4+ CD25+ Immune Regulatory Cells are Required for Induction of Tolerance to Alloantigen *via* Costimulatory Blockade. J Exp Med (2001) 193(11):1311–8. doi: 10.1084/jem.193.11.1311 PMC2193378 11390438

[3] Hara M Kingsley CI Niimi M Read S Turvey SE Bushell AR. IL-10 is Required for Regulatory T Cells to Mediate Tolerance to Alloantigens *In Vivo* . J Immunol (2001) 166(6):3789–96. doi: 10.4049/jimmunol.166.6.3789 11238621

[4] Graca L Thompson S Lin C-Y Adams E Cobbold SP Waldmann H. Both CD4+ CD25+ and CD4+ CD25– Regulatory Cells Mediate Dominant Transplantation Tolerance. J Immunol (2002) 168(11):5558–65. doi: 10.4049/jimmunol.168.11.5558 12023351

[5] Hanash AM Levy RB. Donor CD4+ CD25+ T Cells Promote Engraftment and Tolerance Following MHC-Mismatched Hematopoietic Cell Transplantation. Blood (2005) 105(4):1828–36. doi: 10.1182/blood-2004-08-3213 15494429

[6] Edinger M Hoffmann P Ermann J Drago K Fathman CG Strober S. CD4+ CD25+ Regulatory T Cells Preserve Graft-Versus-Tumor Activity While Inhibiting Graft-Versus-Host Disease After Bone Marrow Transplantation. Nat Med (2003) 9(9):1144–50. doi: 10.1038/nm915 12925844

[7] Trenado A Charlotte F Fisson S Yagello M Klatzmann D Salomon BL. Recipient-Type Specific CD4+ CD25+ Regulatory T Cells Favor Immune Reconstitution and Control Graft-Versus-Host Disease While Maintaining Graft-Versus-Leukemia. J Clin Invest (2003) 112(11):1688–96. doi: 10.1172/JCI17702 PMC281639 14660744

[8] Heinrichs J Li J Nguyen H Wu Y Bastian D Daethanasanmak A. CD8+ Tregs Promote GVHD Prevention and Overcome the Impaired GVL Effect Mediated by CD4+ Tregs in Mice. Oncoimmunology (2016) 5(6):e1146842. doi: 10.1080/2162402x.2016.1146842 27471614 PMC4938369

[9] Brunstein CG Miller JS Cao Q McKenna DH Hippen KL Curtsinger J. Infusion of *Ex Vivo* Expanded T Regulatory Cells in Adults Transplanted With Umbilical Cord Blood: Safety Profile and Detection Kinetics. Blood (2011) 117(3):1061–70. doi: 10.1182/blood-2010-07-293795 PMC3035067 20952687

[10] Brunstein CG Miller JS McKenna DH Hippen KL DeFor TE Sumstad D. Umbilical Cord Blood–Derived T Regulatory Cells to Prevent GVHD: Kinetics, Toxicity Profile, and Clinical Effect. Blood (2016) 127(8):1044–51. doi: 10.1182/blood-2015-06-653667 PMC4768428 26563133

[11] Di Ianni M Falzetti F Carotti A Terenzi A Castellino F Bonifacio E. Tregs Prevent GVHD and Promote Immune Reconstitution in HLA-Haploidentical Transplantation. Blood (2011) 117(14):3921–8. doi: 10.1182/blood-2010-10-311894 21292771

[12] Meyer EH Laport G Xie BJ MacDonald K Heydari K Sahaf B. Transplantation of Donor Grafts With Defined Ratio of Conventional and Regulatory T Cells in HLA-Matched Recipients. JCI Insight (2019) 4(10):e127244. doi: 10.1172/jci.insight.127244 PMC6542607 31092732

[13] Martelli MF Di Ianni M Ruggeri L Falzetti F Carotti A Terenzi A. HLA-Haploidentical Transplantation With Regulatory and Conventional T-Cell Adoptive Immunotherapy Prevents Acute Leukemia Relapse. Blood (2014) 124(4):638–44. doi: 10.1182/blood-2014-03-564401 24923299

[14] Trzonkowski P Bieniaszewska M Juścińska J Dobyszuk A Krzystyniak A Marek N. First-In-Man Clinical Results of the Treatment of Patients With Graft Versus Host Disease With Human *Ex Vivo* Expanded CD4+ CD25+ CD127– T Regulatory Cells. Clin Immunol (2009) 133(1):22–6. doi: 10.1016/j.clim.2009.06.001 19559653

[15] Locafaro G Andolfi G Russo F Cesana L Spinelli A Camisa B. IL-10-Engineered Human CD4(+) Tr1 Cells Eliminate Myeloid Leukemia in an HLA Class I-Dependent Mechanism. Mol Ther (2017) 25(10):2254–69. doi: 10.1016/j.ymthe.2017.06.029 PMC5628869 28807569

[16] Zheng J Liu Y Liu Y Liu M Xiang Z Lam K-T. Human CD8+ Regulatory T Cells Inhibit GVHD and Preserve General Immunity in Humanized Mice. Sci Trans Med (2013) 5(168):168ra9–ra9. doi: 10.1126/scitranslmed.3004943 23325802

[17] Beres AJ Haribhai D Chadwick AC Gonyo PJ Williams CB Drobyski WR. CD8+ Foxp3+ Regulatory T Cells are Induced During Graft-Versus-Host Disease and Mitigate Disease Severity. J Immunol (2012) 189(1):464–74. doi: 10.4049/jimmunol.1200886 PMC3381996 22649199

[18] Grossman WJ Verbsky JW Barchet W Colonna M Atkinson JP Ley TJ. Human T Regulatory Cells can Use the Perforin Pathway to Cause Autologous Target Cell Death. Immunity (2004) 21(4):589–601. doi: 10.1016/j.immuni.2004.09.002 15485635

[19] Grossman WJ Verbsky JW Tollefsen BL Kemper C Atkinson JP Ley TJ. Differential Expression of Granzymes A and B in Human Cytotoxic Lymphocyte Subsets and T Regulatory Cells. Blood (2004) 104(9):2840–8. doi: 10.1182/blood-2004-03-0859 15238416

[20] Fisher GH Rosenberg FJ Straus SE Dale JK Middleton LA Lin AY. Dominant Interfering Fas Gene Mutations Impair Apoptosis in a Human Autoimmune Lymphoproliferative Syndrome. Cell (1995) 81(6):935–46. doi: 10.1016/0092-8674(95)90013-6 7540117

[21] Rieux-Laucat F Le Deist F Hivroz C Roberts IA Debatin KM Fischer A. Mutations in Fas Associated With Human Lymphoproliferative Syndrome and Autoimmunity. Sci (New York NY) (1995) 268(5215):1347–9. doi: 10.1126/science.7539157 7539157

[22] Neven B Magerus-Chatinet A Florkin B Gobert D Lambotte O De Somer L. A Survey of 90 Patients With Autoimmune Lymphoproliferative Syndrome Related to TNFRSF6 Mutation. Blood (2011) 118(18):4798–807. doi: 10.1182/blood-2011-04-347641 21885602

[23] Nabhani S Ginzel S Miskin H Revel-Vilk S Harlev D Fleckenstein B. Deregulation of Fas Ligand Expression as a Novel Cause of Autoimmune Lymphoproliferative Syndrome-Like Disease. Haematologica (2015) 100(9):1189–98. doi: 10.3324/haematol.2014.114967 PMC4800681 26113417

[24] Russell JH Ley TJ. Lymphocyte-Mediated Cytotoxicity. Annu Rev Immunol (2002) 20(1):323–70. doi: 10.1146/annurev.immunol.20.100201.131730 11861606

[25] Arico M Imashuku S Clementi R Hibi S Teramura T Danesino C. Hemophagocytic Lymphohistiocytosis Due to Germline Mutations in SH2D1A, the X-Linked Lymphoproliferative Disease Gene. Blood (2001) 97(4):1131–3. doi: 10.1182/blood.v97.4.1131 11159547

[26] Stepp SE Dufourcq-Lagelouse R Le Deist F Bhawan S Certain S Mathew PA. Perforin Gene Defects in Familial Hemophagocytic Lymphohistiocytosis. Science (1999) 286(5446):1957–9. doi: 10.1126/science.286.5446.1957 10583959

[27] Mahic M Henjum K Yaqub S Bjørnbeth BA Torgersen KM Taskén K. Generation of Highly Suppressive Adaptive CD8+ CD25+ FOXP3+ Regulatory T Cells by Continuous Antigen Stimulation. Eur J Immunol (2008) 38(3):640–6. doi: 10.1002/eji.200737529 18266270

[28] von Boehmer H. Mechanisms of Suppression by Suppressor T Cells. Nat Immunol (2005) 6(4):338–44. doi: 10.1038/ni1180 15785759

[29] Linsley PS Greene JL Brady W Bajorath J Ledbetter JA Peach R. Human B7-1 (CD80) and B7-2 (CD86) Bind With Similar Avidities But Distinct Kinetics to CD28 and CTLA-4 Receptors. Immunity (1994) 1(9):793–801. doi: 10.1016/s1074-7613(94)80021-9 7534620

[30] Hou TZ Qureshi OS Wang CJ Baker J Young SP Walker LS. A Transendocytosis Model of CTLA-4 Function Predicts its Suppressive Behavior on Regulatory T Cells. J Immunol (2015) 194(5):2148–59. doi: 10.4049/jimmunol.1401876 PMC4522736 25632005

[31] Qureshi OS Zheng Y Nakamura K Attridge K Manzotti C Schmidt EM. Trans-Endocytosis of CD80 and CD86: A Molecular Basis for the Cell-Extrinsic Function of CTLA-4. Science (2011) 332(6029):600–3. doi: 10.1126/science.1202947 PMC3198051 21474713

[32] Ng T Britton GJ Hill EV Verhagen J Burton BR Wraith DC. Regulation of Adaptive Immunity; the Role of Interleukin-10. Front Immunol (2013) 4:129. doi: 10.3389/fimmu.2013.00129 23755052 PMC3668291

[33] Kornete M Sgouroudis E Piccirillo CA. ICOS-Dependent Homeostasis and Function of Foxp3+ Regulatory T Cells in Islets of Nonobese Diabetic Mice. J Immunol (2012) 188(3):1064–74. doi: 10.4049/jimmunol.1101303 22227569

[34] Schmitt EG Haribhai D Williams JB Aggarwal P Jia S Charbonnier L-M. IL-10 Produced by Induced Regulatory T Cells (Itregs) Controls Colitis and Pathogenic Ex-Itregs During Immunotherapy. J Immunol (2012) 189(12):5638–48. doi: 10.4049/jimmunol.1200936 PMC3537488 23125413

[35] Palomares O Martin-Fontecha M Lauener R Traidl-Hoffmann C Cavkaytar O Akdis M. Regulatory T Cells and Immune Regulation of Allergic Diseases: Roles of IL-10 and TGF-β. Genes Immun (2014) 15(8):511–20. doi: 10.1038/gene.2014.45 25056447

[36] Boivin GP Ormsby I Jones-Carson J O'toole BA Doetschman T. Germ-Free and Barrier-Raised Tgfβ1-Deficient Mice Have Similar Inflammatory Lesions. Transgenic Res (1997) 6(3):197–202. doi: 10.1023/a:1018490007745 9167267

[37] Diebold RJ Eis MJ Yin M Ormsby I Boivin GP Darrow BJ. Early-Onset Multifocal Inflammation in the Transforming Growth Factor Beta 1-Null Mouse is Lymphocyte Mediated. Proc Natl Acad Sci (1995) 92(26):12215–9. doi: 10.1073/pnas.92.26.12215 PMC40327 8618872

[38] Fantini MC Becker C Monteleone G Pallone F Galle PR Neurath MF. Cutting Edge: TGF-β Induces a Regulatory Phenotype in CD4+ CD25– T Cells Through Foxp3 Induction and Down-Regulation of Smad7. J Immunol (2004) 172(9):5149–53. doi: 10.4049/jimmunol.172.9.5149 15100250

[39] Yadav M Bluestone JA Stephan S. Peripherally Induced Tregs–Role in Immune Homeostasis and Autoimmunity. Front Immunol (2013) 4:232. doi: 10.3389/fimmu.2013.00232 23966994 PMC3736167

[40] Chen W Jin W Hardegen N Lei K-J Li L Marinos N. Conversion of Peripheral CD4+ CD25– Naive T Cells to CD4+ CD25+ Regulatory T Cells by TGF-β Induction of Transcription Factor Foxp3. J Exp Med (2003) 198(12):1875–86. doi: 10.1084/jem.20030152 PMC2194145 14676299

[41] Doisne J-M Bartholin L Yan K-P Garcia CN Duarte N Le Luduec J-B. iNKT Cell Development is Orchestrated by Different Branches of TGF-β Signaling. J Exp Med (2009) 206(6):1365–78. doi: 10.1084/jem.20090127 PMC2715067 19451264

[42] Havenar-Daughton C Li S Benlagha K Marie JC. Development and Function of Murine Rorγt+ iNKT Cells are Under TGF-β Signaling Control. Blood (2012) 119(15):3486–94. doi: 10.1182/blood-2012-01-401604 22371886

[43] Chen M-L Pittet MJ Gorelik L Flavell RA Weissleder R Von Boehmer H. Regulatory T Cells Suppress Tumor-Specific CD8 T Cell Cytotoxicity Through TGF-β Signals *In Vivo* . Proc Natl Acad Sci (2005) 102(2):419–24. doi: 10.1073/pnas.0408197102 PMC544311 15623559

[44] Frimpong-Boateng K van Rooijen N Geiben-Lynn R. Regulatory T Cells Suppress Natural Killer Cells During Plasmid DNA Vaccination in Mice, Blunting the CD8+ T Cell Immune Response by the Cytokine Tgfβ. PloS One (2010) 5(8):e12281. doi: 10.1371/journal.pone.0012281 20808850 PMC2924372

[45] Tran DQ. TGF-β: The Sword, the Wand, and the Shield of FOXP3+ Regulatory T Cells. J Mol Cell Biol (2012) 4(1):29–37. doi: 10.1093/jmcb/mjr033 22158907

[46] Zhong H Liu Y Xu Z Liang P Yang H Zhang X. TGF-β-Induced CD8+ CD103+ Regulatory T Cells Show Potent Therapeutic Effect on Chronic Graft-Versus-Host Disease Lupus by Suppressing B Cells. Front Immunol (2018) 9:35. doi: 10.3389/fimmu.2018.00035 29441062 PMC5797539

[47] Yang X Sun B Wang H Yin C Wang X Ji X. Increased Serum IL-10 in Lupus Patients Promotes Apoptosis of T Cell Subsets *via* the Caspase 8 Pathway Initiated by Fas Signaling. J Biomed Res (2015) 29(3):232. doi: 10.7555/JBR.29.20130037 26060447 PMC4449491

[48] Llorente L Richaud-Patin Y Wijdenes J Alcocer-Varela J Maillot M Durand-Gasselin I. Spontaneous Production of Interleukin-10 by B Lymphocytes and Monocytes in Systemic Lupus Erythematosus. Eur Cytokine Network (1993) 4(6):421–7. 8186374

[49] Llorente L Zou W Levy Y Richaud-Patin Y Wijdenes J Alcocer-Varela J. Role of Interleukin 10 in the B Lymphocyte Hyperactivity and Autoantibody Production of Human Systemic Lupus Erythematosus. J Exp Med (1995) 181(3):839–44. doi: 10.1084/jem.181.3.839 PMC2191898 7869046

[50] Saito I Haruta K Shimuta M Inoue H Sakurai H Yamada K. Fas Ligand-Mediated Exocrinopathy Resembling Sjögren’s Syndrome in Mice Transgenic for IL-10. J Immunol (1999) 162(5):2488–94. 10072487

[51] Schmidt M Lügering N Pauels HG Schulze-Osthoff K Domschke W Kucharzik T. IL-10 Induces Apoptosis in Human Monocytes Involving the CD95 Receptor/Ligand Pathway. Eur J Immunol (2000) 30(6):1769–77. doi: 10.1002/1521-4141(200006)30:6<1769::AID-IMMU1769>3.0.CO;2-9 10898515

[52] Oshima K Sen L Cui G Tung T Sacks BM Arellano-Kruse A. Localized Interleukin-10 Gene Transfer Induces Apoptosis of Alloreactive T Cells *via* FAS/FASL Pathway, Improves Function, and Prolongs Survival of Cardiac Allograft. Transplantation (2002) 73(7):1019–26. doi: 10.1097/00007890-200204150-00002 11965026

[53] Mumm JB Emmerich J Zhang X Chan I Wu L Mauze S. IL-10 Elicits Ifnγ-Dependent Tumor Immune Surveillance. Cancer Cell (2011) 20(6):781–96. doi: 10.1016/j.ccr.2011.11.003 22172723

[54] Rallis KS Corrigan AE Dadah H George AM Keshwara SM Sideris M. Cytokine-Based Cancer Immunotherapy: Challenges and Opportunities for IL-10. Anticancer Res (2021) 41(7):3247–52. doi: 10.21873/anticanres.15110 34230118

[55] Duhen T Duhen R Lanzavecchia A Sallusto F Campbell DJ. Functionally Distinct Subsets of Human FOXP3+ Treg Cells That Phenotypically Mirror Effector Th Cells. Blood (2012) 119(19):4430–40. doi: 10.1182/blood-2011-11-392324 PMC3362361 22438251

[56] Miragaia RJ Gomes T Chomka A Jardine L Riedel A Hegazy AN. Single-Cell Transcriptomics of Regulatory T Cells Reveals Trajectories of Tissue Adaptation. Immunity (2019) 50(2):493–504.e7. doi: 10.1016/j.immuni.2019.01.001 30737144 PMC6382439

[57] Zemmour D Zilionis R Kiner E Klein AM Mathis D Benoist C. Single-Cell Gene Expression Reveals a Landscape of Regulatory T Cell Phenotypes Shaped by the TCR. Nat Immunol (2018) 19(3):291–301. doi: 10.1038/s41590-018-0051-0 29434354 PMC6069633

[58] Höllbacher B Duhen T Motley S Klicznik MM Gratz IK Campbell DJ. Transcriptomic Profiling of Human Effector and Regulatory T Cell Subsets Identifies Predictive Population Signatures. Immunohorizons (2020) 4(10):585–96. doi: 10.4049/immunohorizons.2000037 PMC8085975 33037096

[59] Lichtenheld MG Olsen KJ Lu P Lowrey DM Hameed A Hengartner H. Structure and Function of Human Perforin. Nature (1988) 335(6189):448–51. doi: 10.1038/335448a0 3419519

[60] Law RH Lukoyanova N Voskoboinik I Caradoc-Davies TT Baran K Dunstone MA. The Structural Basis for Membrane Binding and Pore Formation by Lymphocyte Perforin. Nature (2010) 468(7322):447–51. doi: 10.1038/nature09518 21037563

[61] Thiery J Keefe D Boulant S Boucrot E Walch M Martinvalet D. Perforin Pores in the Endosomal Membrane Trigger the Release of Endocytosed Granzyme B Into the Cytosol of Target Cells. Nat Immunol (2011) 12(8):770–7. doi: 10.1038/ni.2050 PMC3140544 21685908

[62] Yannelli J Sullivan J Mandell G Engelhard V. Reorientation and Fusion of Cytotoxic T Lymphocyte Granules After Interaction With Target Cells as Determined by High Resolution Cinemicrography. J Immunol (1986) 136(2):377–82. 3510248

[63] Joosten SA van Meijgaarden KE Savage ND de Boer T Triebel F van der Wal A. Identification of a Human CD8+ Regulatory T Cell Subset That Mediates Suppression Through the Chemokine CC Chemokine Ligand 4. Proc Natl Acad Sci (2007) 104(19):8029–34. doi: 10.1073/pnas.0702257104 PMC1876566 17483450

[64] Salcedo T Azzoni L Wolf SF Perussia B. Modulation of Perforin and Granzyme Messenger RNA Expression in Human Natural Killer Cells. J Immunol (1993) 151(5):2511–20. 8103068

[65] Mellor-Heineke S Villanueva J Jordan MB Marsh R Zhang K Bleesing J. Elevated Granzyme B in Cytotoxic Lymphocytes is a Signature of Immune Activation in Hemophagocytic Lymphohistiocytosis. Front Immunol (2013) 4:72. doi: 10.3389/fimmu.2013.00072 23524976 PMC3605512

[66] Kaiserman D Bird CH Sun J Matthews A Ung K Whisstock JC. The Major Human and Mouse Granzymes are Structurally and Functionally Divergent. J Cell Biol (2006) 175(4):619–30. doi: 10.1083/jcb.200606073 PMC2064598 17116752

[67] Irmler M Hertig S MacDonald HR Sadoul R Becherer J Proudfoot A. Granzyme A is an Interleukin 1 Beta-Converting Enzyme. J Exp Med (1995) 181(5):1917–22. doi: 10.1084/jem.181.5.1917 PMC2191995 7722467

[68] Martinvalet D Dykxhoorn DM Ferrini R Lieberman J. Granzyme A Cleaves a Mitochondrial Complex I Protein to Initiate Caspase-Independent Cell Death. Cell (2008) 133(4):681–92. doi: 10.1016/j.cell.2008.03.032 PMC2840390 18485875

[69] Beresford PJ Kam C-M Powers JC Lieberman J. Recombinant Human Granzyme A Binds to Two Putative HLA-Associated Proteins and Cleaves One of Them. Proc Natl Acad Sci (1997) 94(17):9285–90. doi: 10.1073/pnas.94.17.9285 PMC23158 9256474

[70] Zhu P Zhang D Chowdhury D Martinvalet D Keefe D Shi L. Which Causes Single-Stranded DNA Damage, Targets the Double-Strand Break Repair Protein Ku70. EMBO Rep (2006) 7(4):431–7. doi: 10.1038/sj.embor.7400622 PMC1456912 16440001

[71] Barry M Heibein JA Pinkoski MJ Lee S-F Moyer RW Green DR. Granzyme B Short-Circuits the Need for Caspase 8 Activity During Granule-Mediated Cytotoxic T-Lymphocyte Killing by Directly Cleaving Bid. Mol Cell Biol (2000) 20(11):3781–94. doi: 10.1128/MCB.20.11.3781-3794.2000 PMC85698 10805722

[72] Sutton VR Davis JE Cancilla M Johnstone RW Ruefli AA Sedelies K. Initiation of Apoptosis by Granzyme B Requires Direct Cleavage of Bid, But Not Direct Granzyme B–mediated Caspase Activation. J Exp Med (2000) 192(10):1403–14. doi: 10.1084/jem.192.10.1403 PMC2193191 11085743

[73] Bálint Š Müller S Fischer R Kessler B Harkiolaki M Valitutti S. Supramolecular Attack Particles are Autonomous Killing Entities Released From Cytotoxic T Cells. Science (2020) 368(6493):897–901. doi: 10.1126/science.aay9207 32381591 PMC7116847

[74] Ambrose AR Hazime KS Worboys JD Niembro-Vivanco O Davis DM. Synaptic Secretion From Human Natural Killer Cells is Diverse and Includes Supramolecular Attack Particles. Proc Natl Acad Sci (2020) 117(38):23717–20. doi: 10.1073/pnas.2010274117 PMC7519227 32900953

[75] Liston A Rudensky AY. Thymic Development and Peripheral Homeostasis of Regulatory T Cells. Curr Opin Immunol (2007) 19(2):176–85. doi: 10.1016/j.coi.2007.02.005 17306520

[76] Shevach EM Thornton AM. Ttregs, Ptregs, and Itregs: Similarities and Differences. Immunol Rev (2014) 259(1):88–102. doi: 10.1111/imr.12160 24712461 PMC3982187

[77] Schmidt A Oberle N Krammer PH. Molecular Mechanisms of Treg-Mediated T Cell Suppression. Front Immunol (2012) 3:51. doi: 10.3389/fimmu.2012.00051 22566933 PMC3341960

[78] Vignali DAA Collison LW Workman CJ. How Regulatory T Cells Work. Nat Rev Immunol (2008) 8(7):523–32. doi: 10.1038/nri2343 PMC2665249 18566595

[79] Schmitt EG Williams CB. Generation and Function of Induced Regulatory T Cells. Front Immunol (2013) 4:152. doi: 10.3389/fimmu.2013.00152 23801990 PMC3685796

[80] Zhao DM Thornton AM DiPaolo RJ Shevach EM. Activated CD4+CD25+ T Cells Selectively Kill B Lymphocytes. Blood (2006) 107(10):3925–32. doi: 10.1182/blood-2005-11-4502 PMC1895290 16418326

[81] Gondek DC Lu LF Quezada SA Sakaguchi S Noelle RJ. Cutting Edge: Contact-Mediated Suppression by CD4+CD25+ Regulatory Cells Involves a Granzyme B-Dependent, Perforin-Independent Mechanism. J Immunol (2005) 174(4):1783–6. doi: 10.4049/jimmunol.174.4.1783 15699103

[82] Boissonnas A Scholer-Dahirel A Simon-Blancal V Pace L Valet F Kissenpfennig A. Foxp3+ T Cells Induce Perforin-Dependent Dendritic Cell Death in Tumor-Draining Lymph Nodes. Immunity (2010) 32(2):266–78. doi: 10.1016/j.immuni.2009.11.015 20137985

[83] Gondek DC DeVries V Nowak EC Lu LF Bennett KA Scott ZA. Transplantation Survival is Maintained by Granzyme B+ Regulatory Cells and Adaptive Regulatory T Cells. J Immunol (2008) 181(7):4752–60. doi: 10.4049/jimmunol.181.7.4752 PMC2572718 18802078

[84] Loebbermann J Thornton H Durant L Sparwasser T Webster KE Sprent J. Regulatory T Cells Expressing Granzyme B Play a Critical Role in Controlling Lung Inflammation During Acute Viral Infection. Mucosal Immunol (2012) 5(2):161–72. doi: 10.1038/mi.2011.62 PMC3282434 22236998

[85] Ren X Ye F Jiang Z Chu Y Xiong S Wang Y. Involvement of Cellular Death in TRAIL/DR5-Dependent Suppression Induced by CD4+CD25+ Regulatory T Cells. Cell Death Differentiation (2007) 14(12):2076–84. doi: 10.1038/sj.cdd.4402220 17762882

[86] Deaglio S Dwyer KM Gao W Friedman D Usheva A Erat A. Adenosine Generation Catalyzed by CD39 and CD73 Expressed on Regulatory T Cells Mediates Immune Suppression. J Exp Med (2007) 204(6):1257–65. doi: 10.1084/jem.20062512 PMC2118603 17502665

[87] Pandiyan P Zheng L Ishihara S Reed J Lenardo MJ. CD4+CD25+Foxp3+ Regulatory T Cells Induce Cytokine Deprivation–Mediated Apoptosis of Effector CD4+ T Cells. Nat Immunol (2007) 8(12):1353–62. doi: 10.1038/ni1536 17982458

[88] De La Rosa M Rutz S Dorninger H Scheffold A. Interleukin-2 is Essential for CD4+CD25+ Regulatory T Cell Function. Eur J Immunol (2004) 34(9):2480–8. doi: 10.1002/eji.200425274 15307180

[89] Read S Malmström V Powrie F. Cytotoxic T Lymphocyte-Associated Antigen 4 Plays an Essential Role in the Function of CD25(+)CD4(+) Regulatory Cells That Control Intestinal Inflammation. J Exp Med (2000) 192(2):295–302. doi: 10.1084/jem.192.2.295 10899916 PMC2193261

[90] Tekguc M Wing JB Osaki M Long J Sakaguchi S. Treg-Expressed CTLA-4 Depletes CD80/CD86 by Trogocytosis, Releasing Free PD-L1 on Antigen-Presenting Cells. Proc Natl Acad Sci (2021) 118(30):e2023739118. doi: 10.1073/pnas.2023739118 34301886 PMC8325248

[91] Asseman C Mauze S Leach MW Coffman RL Powrie F. An Essential Role for Interleukin 10 in the Function of Regulatory T Cells That Inhibit Intestinal Inflammation. J Exp Med (1999) 190(7):995–1004. doi: 10.1084/jem.190.7.995 10510089 PMC2195650

[92] Schubert D Bode C Kenefeck R Hou TZ Wing JB Kennedy A. Autosomal Dominant Immune Dysregulation Syndrome in Humans With CTLA4 Mutations. Nat Med (2014) 20(12):1410–6. doi: 10.1038/nm.3746 PMC4668597 25329329

[93] Kuehn HS Ouyang W Lo B Deenick EK Niemela JE Avery DT. Immune Dysregulation in Human Subjects With Heterozygous Germline Mutations in CTLA4. Science (2014) 345(6204):1623–7. doi: 10.1126/science.1255904 PMC4371526 25213377

[94] Sharfe N Dadi HK Shahar M Roifman CM. Human Immune Disorder Arising From Mutation of the α Chain of the Interleukin-2 Receptor. Proc Natl Acad Sci (1997) 94(7):3168–71. doi: 10.1073/pnas.94.7.3168 PMC20340 9096364

[95] Caudy AA Reddy ST Chatila T Atkinson JP Verbsky JW. CD25 Deficiency Causes an Immune Dysregulation, Polyendocrinopathy, Enteropathy, X-Linked–Like Syndrome, and Defective IL-10 Expression From CD4 Lymphocytes. J Allergy Clin Immunol (2007) 119(2):482–7. doi: 10.1016/j.jaci.2006.10.007 17196245

[96] Glocker E-O Kotlarz D Boztug K Gertz EM Schäffer AA Noyan F. Inflammatory Bowel Disease and Mutations Affecting the Interleukin-10 Receptor. New Engl J Med (2009) 361(21):2033–45. doi: 10.1056/NEJMoa0907206 PMC2787406 19890111

[97] Kotlarz D Beier R Murugan D Diestelhorst J Jensen O Boztug K. Loss of Interleukin-10 Signaling and Infantile Inflammatory Bowel Disease: Implications for Diagnosis and Therapy. Gastroenterology (2012) 143(2):347–55. doi: 10.1053/j.gastro.2012.04.045 22549091

[98] Choi BD Gedeon PC Herndon JE Archer GE Reap EA Sanchez-Perez L. Human Regulatory T Cells Kill Tumor Cells Through Granzyme-Dependent Cytotoxicity Upon Retargeting With a Bispecific Antibody. Cancer Immunol Res (2013) 1(3):163. doi: 10.1158/2326-6066.Cir-13-0049 24570975 PMC3932050

[99] Cao X Cai SF Fehniger TA Song J Collins LI Piwnica-Worms DR. Granzyme B and Perforin Are Important for Regulatory T Cell-Mediated Suppression of Tumor Clearance. Immunity (2007) 27(4):635–46. doi: 10.1016/j.immuni.2007.08.014 17919943

[100] Janssens W Carlier V Wu B VanderElst L Jacquemin MG Saint-Remy JM. CD4+CD25+ T Cells Lyse Antigen-Presenting B Cells by Fas-Fas Ligand Interaction in an Epitope-Specific Manner. J Immunol (2003) 171(9):4604–12. doi: 10.4049/jimmunol.171.9.4604 14568934

[101] Choi J Ritchey J Prior JL Holt M Shannon WD Deych E. *In Vivo* Administration of Hypomethylating Agents Mitigate Graft-Versus-Host Disease Without Sacrificing Graft-Versus-Leukemia. Blood (2010) 116(1):129–39. doi: 10.1182/blood-2009-12-257253 PMC2904576 20424188

[102] Brockmann L Gagliani N Steglich B Giannou AD Kempski J Pelczar P. IL-10 Receptor Signaling Is Essential for TR1 Cell Function *In Vivo* . J Immunol (Baltimore Md 1950) (2017) 198(3):1130–41. doi: 10.4049/jimmunol.1601045 PMC5263184 28003377

[103] Roncarolo MG Gregori S Bacchetta R Battaglia M Gagliani N. The Biology of T Regulatory Type 1 Cells and Their Therapeutic Application in Immune-Mediated Diseases. Immunity (2018) 49(6):1004–19. doi: 10.1016/j.immuni.2018.12.001 30566879

[104] Magnani CF Alberigo G Bacchetta R Serafini G Andreani M Roncarolo MG. Killing of Myeloid APCs *via* HLA Class I, CD2 and CD226 Defines a Novel Mechanism of Suppression by Human Tr1 Cells. Eur J Immunol (2011) 41(6):1652–62. doi: 10.1002/eji.201041120 PMC3116154 21469116

[105] Levings MK Sangregorio R Galbiati F Squadrone S de Waal Malefyt R Roncarolo M-G. IFN-α and IL-10 Induce the Differentiation of Human Type 1 T Regulatory Cells. J Immunol (2001) 166(9):5530–9. doi: 10.4049/jimmunol.166.9.5530 11313392

[106] Chatila T Blaeser F Ho N Lederman HM Voulgaropoulos C Helms C. JM2, Encoding a Fork Head-Related Protein, is Mutated in X-Linked Autoimmunity-Allergic Disregulation Syndrome. J Clin Invest (2000) 106(12):75–81. doi: 10.1172/JCI11679 PMC387260 11120765

[107] Bennett CL Christie J Ramsdell F Brunkow ME Ferguson PJ Whitesell L. The Immune Dysregulation, Polyendocrinopathy, Enteropathy, X-Linked Syndrome (IPEX) is Caused by Mutations of FOXP3. Nat Genet (2001) 27(1):20. doi: 10.1038/83713 11137993

[108] Wildin RS Ramsdell F Peake J Faravelli F Casanova J-L Buist N. X-linked neonatal diabetes mellitus, enteropathy and endocrinopathy syndrome is the human equivalent of mouse scurfy. Nat Genet (2001) 27(1):18–20. doi: 10.1038/83707 11137992

[109] Kobayashi I Shiari R Yamada M Kawamura N Okano M Yara A. Novel Mutations of FOXP3 in Two Japanese Patients With Immune Dysregulation, Polyendocrinopathy, Enteropathy, X Linked Syndrome (IPEX). J Med Genet (2001) 38(12):874–6. doi: 10.1136/jmg.38.12.874 PMC1734795 11768393

[110] Zorn E Kim HT Lee SJ Floyd BH Litsa D Arumugarajah S. Reduced Frequency of FOXP3+ CD4+ CD25+ Regulatory T Cells in Patients With Chronic Graft-Versus-Host Disease. Blood (2005) 106(8):2903–11. doi: 10.1182/blood-2005-03-1257 PMC1895303 15972448

[111] Goudy K Aydin D Barzaghi F Gambineri E Vignoli M Mannurita SC. Human IL2RA Null Mutation Mediates Immunodeficiency With Lymphoproliferation and Autoimmunity. Clin Immunol (2013) 146(3):248–61. doi: 10.1016/j.clim.2013.01.004 PMC3594590 23416241

[112] Hsu P Santner-Nanan B Hu M Skarratt K Lee CH Stormon M. IL-10 Potentiates Differentiation of Human Induced Regulatory T Cells *via* STAT3 and Foxo1. J Immunol (2015) 195(8):3665–74. doi: 10.4049/jimmunol.1402898 26363058

[113] Cetica V Sieni E Pende D Danesino C De Fusco C Locatelli F. Genetic Predisposition to Hemophagocytic Lymphohistiocytosis: Report on 500 Patients From the Italian Registry. J Allergy Clin Immunol (2016) 137(1):188–96.e4. doi: 10.1016/j.jaci.2015.06.048 26342526 PMC4699615

[114] Choy JC Hung VH Hunter AL Cheung PK Motyka B Goping IS. Granzyme B Induces Smooth Muscle Cell Apoptosis in the Absence of Perforin: Involvement of Extracellular Matrix Degradation. Arterioscler Thromb Vasc Biol (2004) 24(12):2245–50. doi: 10.1161/01.ATV.0000147162.51930.b7 15472125

[115] Tamzalit F Wang MS Jin W Tello-Lafoz M Boyko V Heddleston JM. Interfacial Actin Protrusions Mechanically Enhance Killing by Cytotoxic T Cells. Sci Immunol (2019) 4(33):eaav5445. doi: 10.1126/sciimmunol.aav5445 30902904 PMC6746172

[116] Bystry RS Aluvihare V Welch KA Kallikourdis M Betz AG. B Cells and Professional APCs Recruit Regulatory T Cells *via* CCL4. Nat Immunol (2001) 2(12):1126–32. doi: 10.1038/ni735 11702067

[117] Lim HW Hillsamer P Banham AH Kim CH. Cutting Edge: Direct Suppression of B Cells by CD4+ CD25+ Regulatory T Cells. J Immunol (2005) 175(7):4180–3. doi: 10.4049/jimmunol.175.7.4180 16177055

[118] Azzi J Skartsis N Mounayar M Magee CN Batal I Ting C. Serine Protease Inhibitor 6 Plays a Critical Role in Protecting Murine Granzyme B-Producing Regulatory T Cells. J Immunol (2013) 191(5):2319–27. doi: 10.4049/jimmunol.1300851 PMC3750098 23913965

[119] Lovo E Zhang M Wang L Ashton-Rickardt PG. Serine Protease Inhibitor 6 is Required to Protect Dendritic Cells From the Kiss of Death. J Immunol (2012) 188(3):1057–63. doi: 10.4049/jimmunol.1102667 PMC3270301 22227570

[120] Zhang M Park SM Wang Y Shah R Liu N Murmann AE. Serine Protease Inhibitor 6 Protects Cytotoxic T Cells From Self-Inflicted Injury by Ensuring the Integrity of Cytotoxic Granules. Immunity (2006) 24(4):451–61. doi: 10.1016/j.immuni.2006.02.002 16618603

[121] Chowdhury D Lieberman J. Death by a Thousand Cuts: Granzyme Pathways of Programmed Cell Death. Annu Rev Immunol (2008) 26:389–420. doi: 10.1146/annurev.immunol.26.021607.090404 18304003 PMC2790083

[122] Karreci ES Eskandari SK Dotiwala F Routray SK Kurdi AT Assaker JP. Human Regulatory T Cells Undergo Self-Inflicted Damage *via* Granzyme Pathways Upon Activation. JCI Insight (2017) 2(21):e91599. doi: 10.1172/jci.insight.91599 PMC5690280 29093262

[123] Efimova OV Kelley TW. Induction of Granzyme B Expression in T-Cell Receptor/CD28-Stimulated Human Regulatory T Cells is Suppressed by Inhibitors of the PI3K-mTOR Pathway. BMC Immunol (2009) 10:59. doi: 10.1186/1471-2172-10-59 19930596 PMC2784757

[124] Toscano MA Bianco GA Ilarregui JM Croci DO Correale J Hernandez JD. Differential Glycosylation of TH1, TH2 and TH-17 Effector Cells Selectively Regulates Susceptibility to Cell Death. Nat Immunol (2007) 8(8):825–34. doi: 10.1038/ni1482 17589510

[125] Gagliani N Magnani CF Huber S Gianolini ME Pala M Licona-Limon P. Coexpression of CD49b and LAG-3 Identifies Human and Mouse T Regulatory Type 1 Cells. Nat Med (2013) 19(6):739–46. doi: 10.1038/nm.3179 23624599

[126] Levings MK Roncarolo MG. T-Regulatory 1 Cells: A Novel Subset of CD4 T Cells With Immunoregulatory Properties. J Allergy Clin Immunol (2000) 106(1 Pt 2):S109–12. doi: 10.1067/mai.2000.106635 10887343

[127] Roncarolo MG Bacchetta R Bordignon C Narula S Levings MK. Type 1 T Regulatory Cells. Immunol Rev (2001) 182:68–79. doi: 10.1034/j.1600-065x.2001.1820105.x 11722624

[128] Akdis M Verhagen J Taylor A Karamloo F Karagiannidis C Crameri R. Immune Responses in Healthy and Allergic Individuals are Characterized by a Fine Balance Between Allergen-Specific T Regulatory 1 and T Helper 2 Cells. J Exp Med (2004) 199(11):1567–75. doi: 10.1084/jem.20032058 PMC2211782 15173208

[129] Tree TI Lawson J Edwards H Skowera A Arif S Roep BO. Naturally Arising Human CD4 T-Cells That Recognize Islet Autoantigens and Secrete Interleukin-10 Regulate Proinflammatory T-Cell Responses *via* Linked Suppression. Diabetes (2010) 59(6):1451–60. doi: 10.2337/db09-0503 PMC2874706 20299476

[130] Cieniewicz B Uyeda MJ Chen PP Sayitoglu EC Liu JM Andolfi G. Engineered Type 1 Regulatory T Cells Designed for Clinical Use Kill Primary Pediatric Acute Myeloid Leukemia Cells. Haematologica (2021) 106(10):2588–97. doi: 10.3324/haematol.2020.263129 PMC8485690 33054128

[131] Nakagawa H Wang L Cantor H Kim HJ. New Insights Into the Biology of CD8 Regulatory T Cells. Adv Immunol (2018) 140:1–20. doi: 10.1016/bs.ai.2018.09.001 30366517

[132] Koh DR Fung-Leung WP Ho A Gray D Acha-Orbea H Mak TW. Less Mortality But More Relapses in Experimental Allergic Encephalomyelitis in CD8-/-Mice. Science (1992) 256(5060):1210–3. doi: 10.1126/science.256.5060.1210 1589800

[133] Jiang H Zhang SL Pernis B. Role of CD8+ T Cells in Murine Experimental Allergic Encephalomyelitis. Science (1992) 256(5060):1213–5. doi: 10.1126/science.256.5060.1213 1375398

[134] Dai H Wan N Zhang S Moore Y Wan F Dai Z. Cutting Edge: Programmed Death-1 Defines CD8+CD122+ T Cells as Regulatory Versus Memory T Cells. J Immunol (2010) 185(2):803–7. doi: 10.4049/jimmunol.1000661 20548035

[135] Liu H Wang Y Zeng Q Zeng YQ Liang CL Qiu F. Suppression of Allograft Rejection by CD8+CD122+PD-1+ Tregs is Dictated by Their Fas Ligand-Initiated Killing of Effector T Cells Versus Fas-Mediated Own Apoptosis. Oncotarget (2017) 8(15):24187–:24195. doi: 10.18632/oncotarget.15551 28445940 PMC5421838

[136] Akane K Kojima S Mak TW Shiku H Suzuki H. CD8+CD122+CD49dlow Regulatory T Cells Maintain T-Cell Homeostasis by Killing Activated T Cells *via* Fas/FasL-Mediated Cytotoxicity. Proc Natl Acad Sci United States America (2016) 113(9):2460–5. doi: 10.1073/pnas.1525098113 PMC4780634 26869716

[137] Zou Q Wu B Xue J Fan X Feng C Geng S. CD8+ Treg Cells Suppress CD8+ T Cell-Responses by IL-10-Dependent Mechanism During H5N1 Influenza Virus Infection. Eur J Immunol (2014) 44(1):103–14. doi: 10.1002/eji.201343583 PMC4165276 24114149

[138] Cosmi L Liotta F Lazzeri E Francalanci M Angeli R Mazzinghi B. Human CD8+CD25+ Thymocytes Share Phenotypic and Functional Features With CD4+CD25+ Regulatory Thymocytes. Blood (2003) 102(12):4107–14. doi: 10.1182/blood-2003-04-1320 12893750

[139] Boor PP Metselaar HJ Jonge SD Mancham S van der Laan LJ Kwekkeboom J. Human Plasmacytoid Dendritic Cells Induce CD8+ LAG-3+ Foxp3+ CTLA-4+ Regulatory T Cells That Suppress Allo-Reactive Memory T Cells. Eur J Immunol (2011) 41(6):1663–74. doi: 10.1002/eji.201041229 21469126

[140] Olson BM Jankowska-Gan E Becker JT Vignali DA Burlingham WJ McNeel DG. Human Prostate Tumor Antigen–Specific CD8+ Regulatory T Cells are Inhibited by CTLA-4 or IL-35 Blockade. J Immunol (2012) 189(12):5590–601. doi: 10.4049/jimmunol.1201744 PMC3735346 23152566

[141] El-Asady R Yuan R Liu K Wang D Gress RE Lucas PJ. TGF-β–Dependent CD103 Expression by CD8+ T Cells Promotes Selective Destruction of the Host Intestinal Epithelium During Graft-Versus-Host Disease. J Exp Med (2005) 201(10):1647–57. doi: 10.1084/jem.20041044 PMC2212926 15897278

[142] Koch SD Uss E Van Lier RA ten Berge IJ. Alloantigen-Induced Regulatory CD8+ CD103+ T Cells. Hum Immunol (2008) 69(11):737–44. doi: 10.1016/j.humimm.2008.08.281 18822329

[143] Uss E Rowshani AT Hooibrink B Lardy NM Van Lier RA ten Berge IJ. CD103 is a Marker for Alloantigen-Induced Regulatory CD8+ T Cells. J Immunol (2006) 177(5):2775–83. doi: 10.4049/jimmunol.177.5.2775 16920912

[144] Saurer L Seibold I Rihs S Vallan C Dumrese T Mueller C. Virus-Induced Activation of Self-Specific Tcrαβ Cd8αα Intraepithelial Lymphocytes Does Not Abolish Their Self-Tolerance in the Intestine. J Immunol (2004) 172(7):4176–83. doi: 10.4049/jimmunol.172.7.4176 15034030

[145] Denning TL Granger S Mucida D Graddy R Leclercq G Zhang W. Mouse Tcrαβ+ Cd8αα Intraepithelial Lymphocytes Express Genes That Down-Regulate Their Antigen Reactivity and Suppress Immune Responses. J Immunol (2007) 178(7):4230–9. doi: 10.4049/jimmunol.178.7.4230 17371979

[146] Poussier P Ning T Banerjee D Julius M. A Unique Subset of Self-Specific Intraintestinal T Cells Maintains Gut Integrity. J Exp Med (2002) 195(11):1491–7. doi: 10.1084/jem.20011793 PMC2193537 12045247

[147] Cai SF Fehniger TA Cao X Mayer JC Brune JD French AR. Differential Expression of Granzyme B and C in Murine Cytotoxic Lymphocytes. J Immunol (2009) 182(10):6287–97. doi: 10.4049/jimmunol.0804333 PMC2714542 19414782

[148] Shires J Theodoridis E Hayday AC. Biological Insights Into Tcrγδ+ and Tcrαβ+ Intraepithelial Lymphocytes Provided by Serial Analysis of Gene Expression (SAGE). Immunity (2001) 15(3):419–34. doi: 10.1016/S1074-7613(01)00192-3 11567632

[149] Bharhani MS Grewal JS Peppler R Enockson C London L London SD. Comprehensive Phenotypic Analysis of the Gut Intra-Epithelial Lymphocyte Compartment: Perturbations Induced by Acute Reovirus 1/L Infection of the Gastrointestinal Tract. Int Immunol (2007) 19(4):567–79. doi: 10.1093/intimm/dxm022 17369189

[150] Bharhani MS Grewal JS Pilgrim MJ Enocksen C Peppler R London L. Reovirus Serotype 1/Strain Lang-Stimulated Activation of Antigen-Specific T Lymphocytes in Peyer’s Patches and Distal Gut-Mucosal Sites: Activation Status and Cytotoxic Mechanisms. J Immunol (2005) 174(6):3580–9. doi: 10.4049/jimmunol.174.6.3580 15749895

[151] Das G Augustine MM Das J Bottomly K Ray P Ray A. An Important Regulatory Role for CD4+ Cd8αα T Cells in the Intestinal Epithelial Layer in the Prevention of Inflammatory Bowel Disease. Proc Natl Acad Sci (2003) 100(9):5324–9. doi: 10.1073/pnas.0831037100 PMC154344 12695566

[152] Sasahara T Tamauchi H Ikewaki N Kubota K. Unique Properties of a Cytotoxic CD4+ CD8+ Intraepithelial T-Cell Line Established From the Mouse Intestinal Epithelium. Microbiol Immunol (1994) 38(3):191–9. doi: 10.1111/j.1348-0421.1994.tb01764.x 8078424

[153] Costes LM Lindenbergh-Kortleve DJ van Berkel LA Veenbergen S Raatgeep HR Simons-Oosterhuis Y. IL-10 Signaling Prevents Gluten-Dependent Intraepithelial CD4+ Cytotoxic T Lymphocyte Infiltration and Epithelial Damage in the Small Intestine. Mucosal Immunol (2019) 12(2):479–90. doi: 10.1038/s41385-018-0118-0 30542112

[154] Najafian N Chitnis T Salama AD Zhu B Benou C Yuan X. Regulatory Functions of CD8+ Cd28–T Cells in an Autoimmune Disease Model. J Clin Invest (2003) 112(7):1037–48. doi: 10.1172/JCI17935 PMC198520 14523041

[155] Manavalan JS Kim-Schulze S Scotto L Naiyer AJ Vlad G Colombo PC. Alloantigen Specific CD8+ CD28– FOXP3+ T Suppressor Cells Induce ILT3+ ILT4+ Tolerogenic Endothelial Cells, Inhibiting Alloreactivity. Int Immunol (2004) 16(8):1055–68. doi: 10.1093/intimm/dxh107 15226269

[156] Strioga M Pasukoniene V Characiejus D. CD8+ CD28– and CD8+ CD57+ T Cells and Their Role in Health and Disease. Immunology (2011) 134(1):17–32. doi: 10.1111/j.1365-2567.2011.03470.x 21711350 PMC3173691

[157] Tsukishiro T Donnenberg AD Whiteside TL. Rapid Turnover of the CD8+ CD28-T-Cell Subset of Effector Cells in the Circulation of Patients With Head and Neck Cancer. Cancer Immunol Immunother (2003) 52(10):599–607. doi: 10.1007/s00262-003-0395-6 12827303 PMC11032778

[158] Fiorentino S Dalod M Olive D Guillet JG Gomard E. Predominant Involvement of CD8+ CD28-Lymphocytes in Human Immunodeficiency Virus-Specific Cytotoxic Activity. J Virol (1996) 70(3):2022–6. doi: 10.1128/jvi.70.3.2022-2026.1996 PMC190033 8627730

[159] Sun Z Zhong W Lu X Shi B Zhu Y Chen L. Association of Graves’ Disease and Prevalence of Circulating IFN-γ-Producing CD28– T Cells. J Clin Immunol (2008) 28(5):464–72. doi: 10.1007/s10875-008-9213-4 18704663

[160] Hamzaoui A Chaouch N Graïri H Ammar J Hamzaoui K. Inflammatory Process of CD8+ CD28– T Cells in Induced Sputum From Asthmatic Patients. Mediators Inflammation (2005) 2005(3):160–6. doi: 10.1155/MI.2005.160 PMC1526471 16106102

[161] Chang CC Ciubotariu R Manavalan JS Yuan J Colovai AI Piazza F. Tolerization of Dendritic Cells by TS Cells: The Crucial Role of Inhibitory Receptors ILT3 and ILT4. Nat Immunol (2002) 3(3):237–43. doi: 10.1038/ni760 11875462

[162] Dai Z Zhang S Xie Q Wu S Su J Li S. Natural CD8+CD122+ T Cells are More Potent in Suppression of Allograft Rejection Than CD4+CD25+ Regulatory T Cells. Am J Transplant (2014) 14(1):39–48. doi: 10.1111/ajt.12515 24219162

[163] Vieyra-Lobato MR Vela-Ojeda J Montiel-Cervantes L López-Santiago R Moreno-Lafont MC. Description of CD8+ Regulatory T Lymphocytes and Their Specific Intervention in Graft-Versus-Host and Infectious Diseases, Autoimmunity, and Cancer. J Immunol Res (2018) 2018. doi: 10.1155/2018/3758713 PMC6098849 30155493

[164] Churlaud G Pitoiset F Jebbawi F Lorenzon R Bellier B Rosenzwajg M. Human and Mouse CD8+ CD25+ FOXP3+ Regulatory T Cells at Steady State and During Interleukin-2 Therapy. Front Immunol (2015) 6:171. doi: 10.3389/fimmu.2015.00171 25926835 PMC4397865

[165] Endharti AT Shi Z Fukuoka Y Nakahara Y Kawamoto Y Takeda K. Cutting Edge: CD8+ CD122+ Regulatory T Cells Produce IL-10 to Suppress IFN-γ Production and Proliferation of CD8+ T Cells. J Immunol (2005) 175(11):7093–7. doi: 10.4049/jimmunol.175.11.7093 16301610

[166] Probst HC McCoy K Okazaki T Honjo T van den Broek M. Resting Dendritic Cells Induce Peripheral CD8+ T Cell Tolerance Through PD-1 and CTLA-4. Nat Immunol (2005) 6(3):280–6. doi: 10.1038/ni1165 15685176

[167] Brimnes J Allez M Dotan I Shao L Nakazawa A Mayer L. Defects in CD8+ Regulatory T Cells in the Lamina Propria of Patients With Inflammatory Bowel Disease. J Immunol (2005) 174(9):5814–22. doi: 10.4049/jimmunol.174.9.5814 15843585

[168] Tennakoon DK Mehta RS Ortega SB Bhoj V Racke MK Karandikar NJ. Therapeutic Induction of Regulatory, Cytotoxic CD8+ T Cells in Multiple Sclerosis. J Immunol (2006) 176(11):7119–29. doi: 10.4049/jimmunol.176.11.7119 16709875

[169] Bresson D Togher L Rodrigo E Chen Y Bluestone JA Herold KC. Anti-CD3 and Nasal Proinsulin Combination Therapy Enhances Remission From Recent-Onset Autoimmune Diabetes by Inducing Tregs. J Clin Invest (2006) 116(5):1371–81. doi: 10.1172/JCI27191 PMC1440705 16628253

[170] Sun D Qin Y Chluba J Epplen JT Wekerle H. Suppression of Experimentally Induced Autoimmune Encephalomyelitis by Cytolytic T–T Cell Interactions. Nature (1988) 332(6167):843–5. doi: 10.1038/332843a0 2965794

[171] Vuddamalay Y Attia M Vicente R Pomié C Enault G Leobon B. Mouse and Human CD8+ CD28low Regulatory T Lymphocytes Differentiate in the Thymus. Immunology (2016) 148(2):187–96. doi: 10.1111/imm.12600 PMC4863570 26924728

[172] Hori S Nomura T Sakaguchi S. Control of Regulatory T Cell Development by the Transcription Factor Foxp3. Science (2003) 299(5609):1057–61. doi: 10.1126/science.1079490 12522256

[173] Rudra D Deroos P Chaudhry A Niec RE Arvey A Samstein RM. Transcription Factor Foxp3 and its Protein Partners Form a Complex Regulatory Network. Nat Immunol (2012) 13(10):1010–9. doi: 10.1038/ni.2402 PMC3448012 22922362

[174] Marson A Kretschmer K Frampton GM Jacobsen ES Polansky JK MacIsaac KD. Foxp3 Occupancy and Regulation of Key Target Genes During T-Cell Stimulation. Nature (2007) 445(7130):931–5. doi: 10.1038/nature05478 PMC3008159 17237765

[175] Bienvenu B Martin B Auffray C Cordier C Bécourt C Lucas B. Peripheral CD8+ CD25+ T Lymphocytes From MHC Class II-Deficient Mice Exhibit Regulatory Activity. J Immunol (2005) 175(1):246–53. doi: 10.4049/jimmunol.175.1.246 15972655

[176] Hahn BH Singh RP La Cava A Ebling FM. Tolerogenic Treatment of Lupus Mice With Consensus Peptide Induces Foxp3-Expressing, Apoptosis-Resistant, Tgfβ-Secreting CD8+ T Cell Suppressors. J Immunol (2005) 175(11):7728–37. doi: 10.4049/jimmunol.175.11.7728 16301683

[177] Singh RP La Cava A Wong M Ebling F Hahn BH. CD8+ T Cell-Mediated Suppression of Autoimmunity in a Murine Lupus Model of Peptide-Induced Immune Tolerance Depends on Foxp3 Expression. J Immunol (2007) 178(12):7649–57. doi: 10.4049/jimmunol.178.12.7649 17548601

[178] Chaput N Louafi S Bardier A Charlotte F Vaillant JC Ménégaux F. Identification of CD8+ CD25+ Foxp3+ Suppressive T Cells in Colorectal Cancer Tissue. Gut (2009) 58(4):520–9. doi: 10.1136/gut.2008.158824 19022917

[179] Aristimuño C de Andrés C Bartolomé M de las Heras V Martínez-Ginés ML Arroyo R. Ifnβ-1a Therapy for Multiple Sclerosis Expands Regulatory CD8+ T Cells and Decreases Memory CD8+ Subset: A Longitudinal 1-Year Study. Clin Immunol (2010) 134(2):148–57. doi: 10.1016/j.clim.2009.09.008 19900844

[180] Safinia N Sagoo P Lechler R Lombardi G. Adoptive Regulatory T Cell Therapy: Challenges in Clinical Transplantation. Curr Opin Organ Transplant (2010) 15(4):427–34. doi: 10.1097/MOT.0b013e32833bfadc 20616725

[181] Safinia N Scotta C Vaikunthanathan T Lechler RI Lombardi G. Regulatory T Cells: Serious Contenders in the Promise for Immunological Tolerance in Transplantation. Front Immunol (2015) 6:438. doi: 10.3389/fimmu.2015.00438 26379673 PMC4553385

[182] Nie J Li YY Zheng SG Tsun A Li B. FOXP3+ Treg Cells and Gender Bias in Autoimmune Diseases. Front Immunol (2015) 6:493. doi: 10.3389/fimmu.2015.00493 26441996 PMC4585344

[183] Correale J Villa A. Isolation and Characterization of CD8+ Regulatory T Cells in Multiple Sclerosis. J Neuroimmunol (2008) 195(1-2):121–34. doi: 10.1016/j.jneuroim.2007.12.004 18234356

[184] Kiniwa Y Miyahara Y Wang HY Peng W Peng G Wheeler TM. CD8+ Foxp3+ Regulatory T Cells Mediate Immunosuppression in Prostate Cancer. Clin Cancer Res (2007) 13(23):6947–58. doi: 10.1158/1078-0432.Ccr-07-0842 18056169

[185] Zheng J Liu Y Qin G Chan PL Mao H Lam KT. Efficient Induction and Expansion of Human Alloantigen-Specific CD8 Regulatory T Cells From Naive Precursors by CD40-Activated B Cells. J Immunol (2009) 183(6):3742–50. doi: 10.4049/jimmunol.0901329 19684082

[186] Tang X Maricic I Purohit N Bakamjian B Reed-Loisel LM Beeston T. Regulation of Immunity by a Novel Population of Qa-1-Restricted CD8αα+ Tcrαβ+ T Cells. J Immunol (2006) 177(11):7645–55. doi: 10.4049/jimmunol.177.11.7645 17114434

[187] Rifa'i M Kawamoto Y Nakashima I Suzuki H. Essential Roles of CD8+ CD122+ Regulatory T Cells in the Maintenance of T Cell Homeostasis. J Exp Med (2004) 200(9):1123–34. doi: 10.1084/jem.20040395 PMC2211869 15520244

[188] Liu Y Lan Q Lu L Chen M Xia Z Ma J. Phenotypic and Functional Characteristic of a Newly Identified CD8+ Foxp3– CD103+ Regulatory T Cells. J Mol Cell Biol (2014) 6(1):81–92. doi: 10.1093/jmcb/mjt026 23861553 PMC3927769

[189] Anz D Mueller W Golic M Kunz WG Rapp M Koelzer VH. CD103 is a Hallmark of Tumor-Infiltrating Regulatory T Cells. Int J Cancer (2011) 129(10):2417–26. doi: 10.1002/ijc.25902 21207371

[190] Lehmann J Huehn J de la Rosa M Maszyna F Kretschmer U Krenn V. Expression of the Integrin αeβ7 Identifies Unique Subsets of CD25+ as Well as CD25– Regulatory T Cells. Proc Natl Acad Sci (2002) 99(20):13031–6. doi: 10.1073/pnas.192162899 PMC130581 12242333

[191] Pauls K Schön M Kubitza RC Homey B Wiesenborn A Lehmann P. Role of Integrin αe (CD103) β7 for Tissue-Specific Epidermal Localization of CD8+ T Lymphocytes. J Invest Dermatol (2001) 117(3):569–75. doi: 10.1046/j.0022-202x.2001.01481.x 11564161

[192] Annacker O Coombes JL Malmstrom V Uhlig HH Bourne T Johansson-Lindbom B. Essential Role for CD103 in the T Cell–Mediated Regulation of Experimental Colitis. J Exp Med (2005) 202(8):1051–61. doi: 10.1084/jem.20040662 PMC2213206 16216886

[193] Hadley G. Role of Integrin CD103 in Promoting Destruction of Renal Allografts by CD8+ T Cells. Am J Transplant (2004) 4(7):1026–32. doi: 10.1111/j.1600-6143.2004.00465.x 15196058

[194] Hadley GA Bartlett ST Via CS Rostapshova EA Moainie S. The epithelial cell-specific integrin, CD103 (alpha E integrin), defines a novel subset of alloreactive CD8+ CTL. J Immunol (1997) 159(8):3748–56. 9378961

[195] Hadley GA Rostapshova EA Gomolka DM Taylor BM Bartlett ST Drachenberg CI. Regulation of the Epithelial Cell-Specific Integrin, CD103, by Human CD8+ Cytolytic T Lymphocytes. Transplantation (1999) 67(11):1418–25. doi: 10.1097/00007890-199906150-00005 10385079

[196] Mei X Li H Zhou X Cheng M Cui K. The Emerging Role of Tissue-Resident Memory CD8+ T Lymphocytes in Human Digestive Tract Cancers. Front Oncol (2021) 11:819505. doi: 10.3389/fonc.2021.819505 35096624 PMC8795735

[197] Jin K Yu Y Zeng H Liu Z You R Zhang H. CD103+ CD8+ Tissue-Resident Memory T Cell Infiltration Predicts Clinical Outcome and Adjuvant Therapeutic Benefit in Muscle-Invasive Bladder Cancer. Br J Cancer (2022), 1–8. doi: 10.1038/s41416-022-01725-6 35165401 PMC9130137

[198] Park CO Kupper TS. The Emerging Role of Resident Memory T Cells in Protective Immunity and Inflammatory Disease. Nat Med (2015) 21(7):688–97. doi: 10.1038/nm.3883 PMC4640452 26121195

[199] Amsen D van Gisbergen KP Hombrink P Van Lier RA. Tissue-Resident Memory T Cells at the Center of Immunity to Solid Tumors. Nat Immunol (2018) 19(6):538–46. doi: 10.1038/s41590-018-0114-2 29777219

[200] Endharti AT Okuno Y Shi Z Misawa N Toyokuni S Ito M. CD8+CD122+ Regulatory T Cells (Tregs) and CD4+ Tregs Cooperatively Prevent and Cure CD4+ Cell-Induced Colitis. J Immunol (2011) 186(1):41–52. doi: 10.4049/jimmunol.1000800 21098236

[201] Wang LX Li Y Yang G Pang PY Haley D Walker EB. CD122+CD8+ Treg Suppress Vaccine-Induced Antitumor Immune Responses in Lymphodepleted Mice. Eur J Immunol (2010) 40(5):1375–85. doi: 10.1002/eji.200839210 PMC3116644 20186876

[202] Mangalam AK Luckey D Giri S Smart M Pease LR Rodriguez M. Two Discreet Subsets of CD8 T Cells Modulate PLP(91-110) Induced Experimental Autoimmune Encephalomyelitis in HLA-DR3 Transgenic Mice. J Autoimmun (2012) 38(4):344–53. doi: 10.1016/j.jaut.2012.02.004 PMC3581307 22459490

[203] Mathews DV Dong Y Higginbotham LB Kim SC Breeden CP Stobert EA. CD122 Signaling in CD8+ Memory T Cells Drives Costimulation-Independent Rejection. J Clin Invest (2018) 128(10):4557–72. doi: 10.1172/JCI95914 PMC6159972 30222140

[204] Zhao Y Cai C Samir J Palgen JL Keoshkerian E Li H. Human CD8 T-Stem Cell Memory Subsets Phenotypic and Functional Characterization are Defined by Expression of CD122 or CXCR3. Eur J Immunol (2021) 51(7):1732–47. doi: 10.1002/eji.202049057 33844287

[205] Zhang Y Joe G Hexner E Zhu J Emerson SG. Host-Reactive CD8+ Memory Stem Cells in Graft-Versus-Host Disease. Nat Med (2005) 11(12):1299–305. doi: 10.1038/nm1326 16288282

[206] Rocha B Vassalli P Guy-Grand D. The V Beta Repertoire of Mouse Gut Homodimeric Alpha CD8+ Intraepithelial T Cell Receptor Alpha/Beta+ Lymphocytes Reveals a Major Extrathymic Pathway of T Cell Differentiation. J Exp Med (1991) 173(2):483–6. doi: 10.1084/jem.173.2.483 PMC2118783 1824858

[207] Eberl G Littman DR. Thymic Origin of Intestinal αß T Cells Revealed by Fate Mapping of Rorγt+ Cells. Science (2004) 305(5681):248–51. doi: 10.1126/science.1096472 15247480

[208] Ruscher R Kummer RL Lee YJ Jameson SC Hogquist KA. Cd8αα Intraepithelial Lymphocytes Arise From Two Main Thymic Precursors. Nat Immunol (2017) 18(7):771–9. doi: 10.1038/ni.3751 PMC5505317 28530714

[209] Neuenhahn M Kerksiek KM Nauerth M Suhre MH Schiemann M Gebhardt FE. Cd8α+ Dendritic Cells are Required for Efficient Entry of Listeria Monocytogenes Into the Spleen. Immunity (2006) 25(4):619–30. doi: 10.1016/j.immuni.2006.07.017 17027298

[210] Gangadharan D Lambolez F Attinger A Wang-Zhu Y Sullivan BA Cheroutre H. Identification of Pre-and Postselection Tcrαβ+ Intraepithelial Lymphocyte Precursors in the Thymus. Immunity (2006) 25(4):631–41. doi: 10.1016/j.immuni.2006.08.018 17045820

[211] Yamagata T Mathis D Benoist C. Self-Reactivity in Thymic Double-Positive Cells Commits Cells to a CD8αα Lineage With Characteristics of Innate Immune Cells. Nat Immunol (2004) 5(6):597–605. doi: 10.1038/ni1070 15133507

[212] Kim H-J Verbinnen B Tang X Lu L Cantor H. Inhibition of Follicular T-Helper Cells by CD8+ Regulatory T Cells is Essential for Self Tolerance. Nature (2010) 467(7313):328–32. doi: 10.1038/nature09370 PMC3395240 20844537

[213] Kim H-J Wang X Radfar S Sproule TJ Roopenian DC Cantor H. CD8+ T Regulatory Cells Express the Ly49 Class I MHC Receptor and are Defective in Autoimmune Prone B6-Yaa Mice. Proc Natl Acad Sci (2011) 108(5):2010–5. doi: 10.1073/pnas.1018974108 PMC3033298 21233417

[214] Rahim MM Tu MM Mahmoud AB Wight A Abou-Samra E Lima PD. Ly49 Receptors: Innate and Adaptive Immune Paradigms. Front Immunol (2014) 5:145. doi: 10.3389/fimmu.2014.00145 24765094 PMC3980100

[215] Bubier JA Bennett SM Sproule TJ Lyons BL Olland S Young DA. Treatment of BXSB-Yaa Mice With IL-21r-Fc Fusion Protein Minimally Attenuates Systemic Lupus Erythematosus. Ann New York Acad Sci (2007) 1110(1):590–601. doi: 10.1196/annals.1423.063 17911475

[216] Saligrama N Zhao F Sikora MJ Serratelli WS Fernandes RA Louis DM. Opposing T Cell Responses in Experimental Autoimmune Encephalomyelitis. Nature (2019) 572(7770):481–7. doi: 10.1038/s41586-019-1467-x PMC7145319 31391585

[217] Dissen E Fossum S Hoelsbrekken SE Saether PC. NK Cell Receptors in Rodents and Cattle. Semin Immunol (2008) 20(6):369–75. doi: 10.1016/j.smim.2008.09.007 18977671

[218] Middleton D Gonzelez F. The Extensive Polymorphism of KIR Genes. Immunology (2010) 129(1):8–19. doi: 10.1111/j.1365-2567.2009.03208.x 20028428 PMC2807482

[219] Li J Zaslavsky M Su Y Guo J Sikora MJ van Unen V. KIR+ CD8+ T Cells Suppress Pathogenic T Cells and are Active in Autoimmune Diseases and COVID-19. Science (2022):eabi9591. doi: 10.1126/science.abi9591 35258337 PMC8995031

[220] Cai SF Cao X Hassan A Fehniger TA Ley TJ. Granzyme B is Not Required for Regulatory T Cell–Mediated Suppression of Graft-Versus-Host Disease. Blood (2010) 115(9):1669–77. doi: 10.1182/blood-2009-07-233676 PMC2832813 19965675

[221] Zhang X Ouyang X Xu Z Chen J Huang Q Liu Y. CD8+ CD103+ Itregs Inhibit Chronic Graft-Versus-Host Disease With Lupus Nephritis by the Increased Expression of CD39. Mol Ther (2019) 27(11):1963–73. doi: 10.1016/j.ymthe.2019.07.014 PMC6838901 31402273

[222] Deng W Xu M Meng Q Li Z Qiu X Yin S. CD8+ CD103+ Itregs Inhibit the Progression of Lupus Nephritis by Attenuating Glomerular Endothelial Cell Injury. Rheumatology (2019) 58(11):2039–50. doi: 10.1093/rheumatology/kez112 31329981

[223] Sagoo P Ali N Garg G Nestle FO Lechler RI Lombardi G. uman Regulatory T Cells With Alloantigen Specificity are More Potent Inhibitors of Alloimmune Skin Graft Damage Than Polyclonal Regulatory T Cells. Sci Transl Med (2011) 3(83):83ra42. doi: 10.1126/scitranslmed.3002076 PMC3776382 21593402

[224] Zhang Q Lu W Liang CL Chen Y Liu H Qiu F. Chimeric Antigen Receptor (CAR) Treg: A Promising Approach to Inducing Immunological Tolerance. Front Immunol (2018) 9:2359. doi: 10.3389/fimmu.2018.02359 30369931 PMC6194362

[225] Mohseni YR Tung SL Dudreuilh C Lechler RI Fruhwirth GO Lombardi G. The Future of Regulatory T Cell Therapy: Promises and Challenges of Implementing CAR Technology. Front Immunol (2020) 11:1608(1608). doi: 10.3389/fimmu.2020.01608 32793236 PMC7393941

[226] Sadelain M Brentjens R Rivière I. The Basic Principles of Chimeric Antigen Receptor Design. Cancer Discovery (2013) 3(4):388–98. doi: 10.1158/2159-8290.Cd-12-0548 PMC3667586 23550147

[227] Dawson NAJ Levings MK. Antigen-Specific Regulatory T Cells: Are Police CARs the Answer? Trans Res (2017) 187:53–8. doi: 10.1016/j.trsl.2017.06.009 28688236

[228] MacDonald KG Hoeppli RE Huang Q Gillies J Luciani DS Orban PC. Alloantigen-Specific Regulatory T Cells Generated With a Chimeric Antigen Receptor. J Clin Invest (2016) 126(4):1413–24. doi: 10.1172/JCI82771 PMC4811124 26999600

[229] Bézie S Charreau B Vimond N Lasselin J Gérard N Nerrière-Daguin V. Human CD8+ Tregs Expressing a MHC-Specific CAR Display Enhanced Suppression of Human Skin Rejection and GVHD in NSG Mice. Blood Adv (2019) 3(22):3522–38. doi: 10.1182/bloodadvances.2019000411 PMC6880915 31730699

[230] Noyan F Zimmermann K Hardtke-Wolenski M Knoefel A Schulde E Geffers R. Prevention of Allograft Rejection by Use of Regulatory T Cells With an MHC-Specific Chimeric Antigen Receptor. Am J Transplant (2017) 17(4):917–30. doi: 10.1111/ajt.14175 27997080

[231] Yoon J Schmidt A Zhang AH Königs C Kim YC Scott DW. FVIII-Specific Human Chimeric Antigen Receptor T-Regulatory Cells Suppress T- and B-Cell Responses to FVIII. Blood (2017) 129(2):238–45. doi: 10.1182/blood-2016-07-727834 PMC5234219 28064157

[232] Boardman DA Philippeos C Fruhwirth GO Ibrahim MA Hannen RF Cooper D. Smyth LA.Expression of a Chimeric Antigen Receptor Specific for Donor HLA Class I Enhances the Potency of Human Regulatory T Cells in Preventing Human Skin Transplant Rejection. Am J Transplant (2017) 17(4):931–43. doi: 10.1111/ajt.14185 28027623

[233] Imura Y Ando M Kondo T Ito M Yoshimura A. CD19-Targeted CAR Regulatory T Cells Suppress B Cell Pathology Without GvHD. JCI Insight (2020) 5(14):e136185. doi: 10.1172/jci.insight.136185 PMC7453900 32525846

[234] Boroughs AC Larson RC Choi BD Bouffard AA Riley LS Schiferle E. Chimeric Antigen Receptor Costimulation Domains Modulate Human Regulatory T Cell Function. JCI Insight (2019) 5(8):e126194. doi: 10.1172/jci.insight.126194 PMC6538349 30869654

[235] Koristka S Kegler A Bergmann R Arndt C Feldmann A Albert S. Engrafting Human Regulatory T Cells With a Flexible Modular Chimeric Antigen Receptor Technology. J Autoimmun (2018) 90:116–31. doi: 10.1016/j.jaut.2018.02.006 29503042

[236] Dawson NAJ Rosado-Sánchez I Novakovsky GE Fung VCW Huang Q McIver E. Functional Effects of Chimeric Antigen Receptor Co-Receptor Signaling Domains in Human Regulatory T Cells. Sci Trans Med (2020) 12(557):eaaz3866. doi: 10.1126/scitranslmed.aaz3866 32817364

[237] Kansal R Richardson N Neeli I Khawaja S Chamberlain D Ghani M. Sustained B Cell Depletion by CD19-Targeted CAR T Cells is a Highly Effective Treatment for Murine Lupus. Sci Transl Med (2019) 11(482):eaav1648. doi: 10.1126/scitranslmed.aav1648 30842314 PMC8201923

[238] Pennell CA Barnum JL McDonald-Hyman CS Panoskaltsis-Mortari A Riddle MJ Xiong Z. Human CD19-Targeted Mouse T Cells Induce B Cell Aplasia and Toxicity in Human CD19 Transgenic Mice. Mol Ther (2018) 26(6):1423–34. doi: 10.1016/j.ymthe.2018.04.006 PMC5986973 29735365

[239] Siegler EL Kenderian SS. Neurotoxicity and Cytokine Release Syndrome After Chimeric Antigen Receptor T Cell Therapy: Insights Into Mechanisms and Novel Therapies. Front Immunol (2020) 11:1973. doi: 10.3389/fimmu.2020.01973 32983132 PMC7485001

[240] Elinav E Waks T Eshhar Z. Redirection of Regulatory T Cells With Predetermined Specificity for the Treatment of Experimental Colitis in Mice. Gastroenterology (2008) 134(7):2014–24. doi: 10.1053/j.gastro.2008.02.060 18424268

[241] Bonifant CL Jackson HJ Brentjens RJ Curran KJ. Toxicity and Management in CAR T-Cell Therapy. Mol Ther - Oncolytics (2016) 3:16011. doi: 10.1038/mto.2016.11 27626062 PMC5008265

